# Meteorological factors, population immunity, and COVID-19 incidence: A global multi-city analysis

**DOI:** 10.1097/EE9.0000000000000338

**Published:** 2024-11-11

**Authors:** Denise Feurer, Tim Riffe, Maxi Stella Kniffka, Enrique Acosta, Ben Armstrong, Malcolm Mistry, Rachel Lowe, Dominic Royé, Masahiro Hashizume, Lina Madaniyazi, Chris Fook Sheng Ng, Aurelio Tobias, Carmen Íñiguez, Ana Maria Vicedo-Cabrera, Martina S. Ragettli, Eric Lavigne, Patricia Matus Correa, Nicolás Valdés Ortega, Jan Kyselý, Aleš Urban, Hans Orru, Ene Indermitte, Marek Maasikmets, Marco Dallavalle, Alexandra Schneider, Yasushi Honda, Barrak Alahmad, Antonella Zanobetti, Joel Schwartz, Gabriel Carrasco, Iulian Horia Holobâca, Ho Kim, Whanhee Lee, Michelle L. Bell, Noah Scovronick, Fiorella Acquaotta, Micheline de Sousa Zanotti Stagliorio Coélho, Magali Hurtado Diaz, Eunice Elizabeth Félix Arellano, Paola Michelozzi, Massimo Stafoggia, Francesca de’Donato, Shilpa Rao, Francesco Di Ruscio, Xerxes Seposo, Yuming Guo, Shilu Tong, Pierre Masselot, Antonio Gasparrini, Francesco Sera

**Affiliations:** a Unit of Biostatistics, Epidemiology and Public Health (UBEP), University of Padua, Padua, Italy; b Interdepartmental Research Center of Geomatics (CIRGEO), University of Padua, Padua, Italy; c Universidad del País Vasco (UPV/EHU), Leioa, Spain; d Ikerbasque (Basque Foundation for Science), Bilbao, Spain; e Max Planck Institute for Demographic Research, Rostock, Germany; f Universität Rostock, Germany; g Centre d’Estudis Demogràfics, Bellaterra, Spain; h Department of Public Health, Environments and Society, London School of Hygiene & Tropical Medicine, London, UK; i Environment & Health Modelling (EHM) Lab, Department of Public Health, Environments and Society, London School of Hygiene & Tropical Medicine, London, UK; j Department of Economics, Ca’ Foscari University of Venice, Venice, Italy; k Barcelona Supercomputing Center (BSC), Barcelona, Spain; l Catalan Institution for Research and Advanced Studies (ICREA), Barcelona, Spain; m Centre on Climate Change & Planetary Health and Centre for Mathematical Modelling of Infectious Diseases, London School of Hygiene & Tropical Medicine, London, UK; n CIBERESP, Madrid. Spain; o Climate Research Foundation (FIC), Madrid, Spain; p School of Tropical Medicine and Global Health, Nagasaki University, Japan; q Department of Global Health Policy, Graduate School of Medicine, The University of Tokyo, Tokyo, Japan; r Institute of Environmental Assessment and Water Research (IDAEA), Spanish Council for Scientific Research (CSIC), Barcelona, Spain; s Department of Statistics and Computational Research. Universitat de València, València, Spain; t Institute of Social and Preventive Medicine, University of Bern, Bern, Switzerland; u Oeschger Center for Climate Change Research, University of Bern, Bern, Switzerland; v Swiss Tropical and Public Health Institute, Allschwil, Switzerland; w University of Basel, Basel, Switzerland; x School of Epidemiology and Public Health, Faculty of Medicine, University of Ottawa, Ottawa, Canada; y Environmental Health Science and Research Bureau, Health Canada, Ottawa, Canada; z Department of Public Health, Universidad de los Andes, Santiago, Chile; aa Institute of Atmospheric Physics of the Czech Academy of Sciences, Prague, Czech Republic; ab Faculty of Environmental Sciences, Czech University of Life Sciences, Prague, Czech Republic; ac Institute of Family Medicine and Public Health, University of Tartu, Tartu, Estonia; ad Estonian Environmental Research Centre, Tallinn, Estonia; ae Institute of Epidemiology, Helmholtz Zentrum München – German Research Center for Environmental Health (GmbH), Neuherberg, Germany; af Center for Climate Change Adaptation, National Institute for Environmental Studies, Tsukuba, Japan; ag Faculty of Health and Sport Sciences, University of Tsukuba, Tsukuba, Japan; ah Department of Environmental Health, Harvard T.H. Chan School of Public Health, Harvard University, Boston, USA; ai Institute of Tropical Medicine “Alexander von Humboldt,” Universidad Peruana Cayetano Heredia, Lima, Peru; aj Faculty of Geography, Babes-Bolyai University, Cluj-Napoca, Romania; ak Department of Public Health Science, Graduate School of Public Health, & Institute of Health and Environment, Seoul National University, Seoul, Republic of Korea; al School of Biomedical Convergence Engineering, Pusan National University; am School of the Environment, Yale University, New Haven, CT, USA; an Korea University, Seoul, South Korea; ao Department of Environmental Health. Rollins School of Public Health, Emory University, Atlanta, USA; ap Department of Earth Sciences, University of Torino, Italy; aq Institute of Advanced Studies, University of São Paulo, São Paulo, Brazil; ar Department of Environmental Health, National Institute of Public Health, Cuernavaca, Morelos, Mexico; as Department of Epidemiology, Lazio Regional Health Service, Rome, Italy; at Norwegian Institute of Public Health, Oslo, Norway; au Department of Hygiene, Graduate School of Medicine, Hokkaido University, Sapporo, Japan; av Department of Epidemiology and Preventive Medicine, School of Public Health and Preventive Medicine, Monash University, Melbourne, Australia; aw Climate, Air Quality Research Unit, School of Public Health and Preventive Medicine, Monash University, Melbourne, Australia; ax National Institute of Environmental Health, China CDC, Beijing, China; ay School of Public Health and Social Work, Queensland University of Technology, Brisbane, Australia; az Department of Statistics, Computer Science and Applications “G. Parenti,” University of Florence, Florence, Italy

**Keywords:** Temperature, Humidity, Solar radiation, Precipitation, COVID-19, Multi-Country Multi-City Collaborative Research Network, Time-series design, Distributed lag nonlinear models

## Abstract

**Objectives:**

While COVID-19 continues to challenge the world, meteorological variables are thought to impact COVID-19 transmission. Previous studies showed evidence of negative associations between high temperature and absolute humidity on COVID-19 transmission. Our research aims to fill the knowledge gap on the modifying effect of vaccination rates and strains on the weather-COVID-19 association.

**Methods:**

Our study included COVID-19 data from 439 cities in 22 countries spanning 3 February 2020 – 31 August 2022 and meteorological variables (temperature, relative humidity, absolute humidity, solar radiation, and precipitation). We used a two-stage time-series design to assess the association between meteorological factors and COVID-19 incidence. For the exposure modeling, we used distributed lag nonlinear models with a lag of up to 14 days. Finally, we pooled the estimates using a random effect meta-analytic model and tested vaccination rates and dominant strains as possible effect modifiers.

**Results:**

Our results showed an association between temperature and absolute humidity on COVID-19 transmission. At 5 °C, the relative risk of COVID-19 incidence is 1.22-fold higher compared to a reference level at 17 °C. Correlated with temperature, we observed an inverse association for absolute humidity. We observed a tendency of increased risk on days without precipitation, but no association for relative humidity and solar radiation. No interaction between vaccination rates or strains on the weather-COVID-19 association was observed.

**Conclusions:**

This study strengthens previous evidence of a relationship of temperature and absolute humidity with COVID-19 incidence. Furthermore, no evidence was found that vaccinations and strains significantly modify the relationship between environmental factors and COVID-19 transmission.

**What this study adds**

This study seeks to add to the existing literature on the associations between meteorological factors and COVID-19 transmission by considering the possible effects of temperature, humidity, solar radiation, and precipitation over a large and heterogenous set of cities, with a longer time series and fine granular timescale allowing for more robust statistical inference than earlier studies with similar designs. A novelty of this study is that we additionally assessed the modifying effects of vaccination rates and dominant strains.

## Introduction

The COVID-19 pandemic represents the most significant public health crisis in recent years. Years after the first cases emerged, the world is still recovering from its impacts. The pandemic has not only taken its toll on healthcare systems worldwide but has brought unprecedented political, social, and economic challenges.^1,2^ While the initial waves of cases and government shutdowns are over, new strains are emerging, leading to new outbreaks. As the pandemic is turning endemic, the impacts continue to pressure society to react and adapt.^3^ The continuing impacts of COVID-19 are still mounting; hence, it is key to gain a deeper understanding of SARS-CoV-19 to reduce its impact, thereby minimizing further strain on governments and healthcare systems.

Human mobility and government stringency have been shown to substantially affect the spread of the disease.^4^ SARS-CoV-19 is mainly airborne, and an individual can be infected by breathing in contaminated air. Actions such as social distancing, wearing masks, and hand disinfection can significantly reduce the spread of disease.^5^ A review of existing evidence found that social health factors were strongly related to the COVID-19 spread.^6^ The natural environment, however, has been hypothesized to impact the spread as well.^7,8^

Since other respiratory diseases are often seasonal and are therefore affected directly or indirectly by the environment, it has been postulated that SARS-CoV-19 could behave similarly.^2^ The World Meteorological Organization published a report, confirming that both meteorology and air quality played a secondary part in the transmission of COVID-19.^9^ Several studies have shown that temperature and absolute humidity (AH) indeed have some impact on the disease transmission; one study hypothesized that humid climates and summer weather would make outbreaks more likely.^6,8,10–12^ However, other studies found that high temperatures and humidity were associated with decreased incidence of COVID-19.^4,10,11^ Another study found a small positive association between ultraviolet (UV) radiation and COVID-19 incidence.^11^ Many other studies were inconclusive or did not show significant impacts, which might be due to the analysis on only data from early in the pandemic.^4,6^ Some researchers speculate that during the pandemic stage of a new virus, environmental impacts play a minor role in a virus’ spread but that these might become more important factors during endemic infections.^8,10^

While several studies have analyzed environmental predictors or determinants of COVID-19, we still lack a comprehensive understanding about key determinants of the COVID-19 spread. Many studies may be limited due to either having had a short timeframe or a focus on a single city or country. Our study aims to fill this research gap by including data from 439 cities in 22 countries and a timeframe spanning multiple waves from 3 February 2020 until 31 August 2022. Understanding the factors impacting the spread of the virus is one of the main tools we have to inform policymakers and citizens for the effective control of COVID-19 and its related impacts, and such knowledge is also critical for accurate estimates of impacts under various conditions.

This study seeks to add to the existing literature on this topic by considering the possible effects of temperature, humidity, solar radiation, and precipitation over a large and heterogenous set of cities, with a longer time series and fine granular timescale allowing for a more robust statistical inference than earlier studies with similar designs. A novelty of this study is that we additionally assessed the modifying effects of vaccination rates and dominant strains.

## Methods

### Data sources and extraction

#### COVID-19 data

##### Data extraction

The data for this study was retrieved from public sources and integrated with data from the Multi-Country Multi-City (MCC) Collaborative Research Network (https://mccstudy.lshtm.ac.uk/). The full list of COVID-19 data sources can be found in the supplemental material (Table S1; http://links.lww.com/EE/A311). We initially considered data of COVID-19 daily incidence from the start of the pandemic until 31 August 2022 in 458 cities and 22 countries. We included all the cities that are part of the MCC network, as we have contextual data for those cities available. We only excluded those that did not have daily COVID-19 data available.

##### Data cleaning and processing

We processed each city’s time series of daily COVID-19 cases to remove outliers and implausible values. In the first step, negative values and outliers were set to missing values, whereas outliers were defined as values above the threshold, set as 10 times the interquartile range (IQR); the IQR was calculated over days with more than 10 cases. This step was followed by setting isolated zeros to missing values. That is, days with zero cases whose surrounding 5-day average cases were greater than zero were set to missing. Finally, missing values were imputed using a 5-day centered moving average. To exclude the first imported cases, the location-specific time series’ were shortened to start up to 14 days (depending on the days of lag considered) before the first time a city counted the COVID-19 incidence of 10 cases.

Further visual checks were performed, and time series with >5% missing values and unexplainable spikes were excluded. After the data cleaning and winnowing process, we considered the COVID-19 daily time series for 439 cities in 22 countries.

#### Meteorological variables

Meteorological variables selected for the analysis included: mean temperature, AH, relative humidity (RH), precipitation, and solar radiation. We retrieved this meteorological data from the Copernicus ERA-5 Land dataset with a latitude-longitude grid size of 0.1° × 0.1°, roughly translating to a 9 × 9 km grid.^13^ We extracted daily averages for temperature and dew temperature (2 m above the surface), surface solar radiation, total precipitation, and surface pressure from the grid cell containing the centroid of each given city. We calculated RH and AH from temperature, dew temperature, and surface pressure using the R “humidity” package.^14^

#### Government interventions

We used data on the Government Stringency Index (GSI) from the Oxford COVID-19 Government Response Tracker (OxCGRT) to control for the changing governmental public health measures implemented in response to the pandemic, which may play a role in modulating the association between COVID-19 incidence and environmental factors.^15^ The GSI scale ranges from zero to 100 points, where a score of 100 represents the strictest policy measures to slow down the transmission. Policy measures include interventions such as closures, movement restrictions, income support, and testing policies.

#### Vaccination

We retrieved vaccination data for each country from https://OurWorldinData.org.^16^ State-specific vaccination data for the USA, was additionally retrieved from the Johns Hopkins University to account for large variability in vaccination coverage between the different states.^9^ A person was considered vaccinated when they received at least one dose of vaccination, and fully vaccinated, when they received all the doses as prescribed by the initial vaccination protocol (1–2 doses depending on the vaccine). Each measure is defined as the number of people vaccinated, or fully vaccinated, respectively, per 100 people over the country’s total population. The main analysis considered the countries/periods with less than 60% and periods with more than 60% vaccination coverage.

#### Strains

We also considered the dominant strain for each period in the analysis. We retrieved this information from online public sources.^17^ The main strains used in the analysis were defined as Delta, Omicron, and “Initial.” Hereafter, “Initial” is referred to period of the first wave, when there was limited strain testing, and several mutant strains emerged before any specific strains became the dominant drivers of the pandemic. The dominant strain was defined as the strain representing the majority of cases per country for each month.

### Statistical analysis

#### Descriptive analysis

Daily new and cumulative COVID-19 cases for each country were aggregated from the included cities data. We calculated cases per 100,000 inhabitants using the total population size of each city.^18^ Daily time series of the meteorological variables (mean temperature, relative and AH, solar radiation, and precipitation), government interventions (GSI), and vaccination (total vaccinated per 100 people in the total population of the country) were computed for each country for the entire observation period.

#### Two-stage design

We used a two-stage design to assess the association and temporal variation between meteorological factors and COVID-19 incidence. In the first stage, we estimated the city-specific exposure-response associations, considering time-varying confounding in a time-series regression. We used a meta-analytical model in the second stage to combine city-specific estimates to obtain the pooled exposure-response association curve.

The first step in the first stage was to create independent models for each exposure and location. We used a Generalized Linear Model with a quasi-Poisson distribution to model the case counts. We modeled the exposures using distributed lag nonlinear models.^19^ We defined a 3rd degree polynomial for the basis function for the exposure dimension (temperature, AH, RH, solar radiation, and precipitation). We modeled the lag dimension using a natural cubic spline with two equally spaced (at logarithmic scale) internal knots. We used a lag of 14 days for the main analyses, due to the estimated incubation period of 6 days for COVID-19 and a delay in testing or reporting.^20,21^ We defined a bi-dimensional basis called a “cross-basis” by combining the two previously created bases.^22^

We considered several confounding factors for the main model. We included a series of dummy day-of-week variable (dow), since various factors, such as reporting, social behavior, and testing capacity, are known to vary among weekdays, in addition to the heterogeneous definitions of case date among sources. We also considered other time-vary confounders, such as temporal trend, modeled with a natural spline function of the date with 10 degrees of freedom (df), and governmental interventions, modeled with a linear lag association model of GSI considering up to 14 days of lag dependence. We built the model using the R package “dlnm.”^23^

To evaluate the independent effect of temperature, we fitted four time-series models with temperature and each of the other exposure variables (AH, RH, solar radiation, and precipitation).

For each meteorological variable, we estimated the temporal variation of the relationships with COVID-19 using distributed lag nonlinear models, expressed through an interaction between meteorological variables and flexibly defined periods for each city. The time periods were defined by vaccination coverage (below and above 60%) and by the dominant strain (Initial, Delta, or Omicron).

For the subsequent second-stage meta-analysis, we used the R package “mixmeta.”^24^ The coefficients, representing the estimated meteorology-COVID-19 associations cumulated over all lags, and their covariance matrix, obtained at the first step, were pooled from all included locations using a random effect meta-analytic model. Groups defined jointly by country and climatic zones were considered as a random effect in the main model. Using the pooled polynomial basis coefficients, the pooled mean curve of COVID-19 risk was plotted against each exposure and expressed as relative risk (RR) with median value over all cities set at the baseline.

All the data and code examples used in the analyses will be available on request.

#### Sensitivity analysis

We performed sensitivity analysis considering a linear association for meteorological variables and COVID-19. Additionally, we performed sensitivity analysis considering different thresholds for vaccination coverage (e.g., 75%) and considering fully vaccinated populations. We also considered seasonality by analyzing the associations during winter months and summer months separately. We defined summer months as June, July, and August and winter months as December, January, and February for the northern hemisphere and the reversed months for the southern hemisphere. These intra-annual and trend effects were estimated with an interaction term between natural spline parametrization of the day of the year (with 3 degrees of freedom) and year considered as a categorical variable.

## Results

### Descriptive

**Figure 1.**
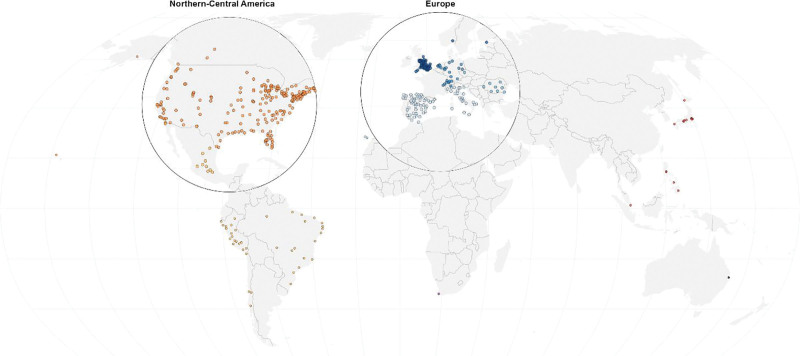
Map of cities included in the analysis, with the majority of data coming from North America, Europe, and Latin America. Additional cities included from South Africa, Australia, and Asia.

**Figure 2.**
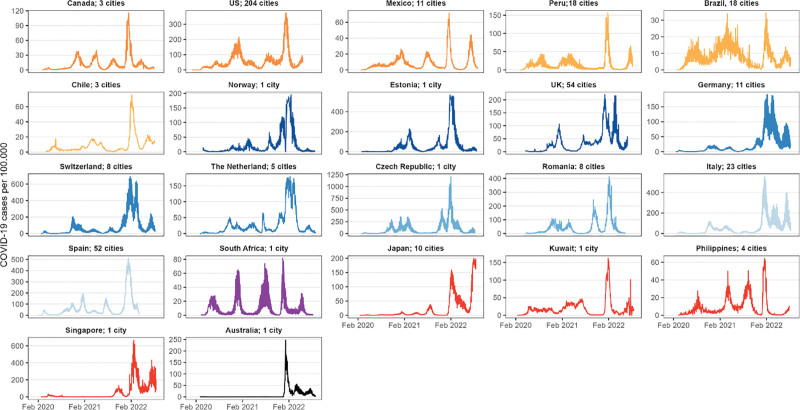
Time series of COVID-19 cases per 100,000 inhabitants aggregated by country, with number of included cities ranging from 1 (e.g., Kuwait) to 204 (USA).

For the final analysis, we considered 96.1 million confirmed COVID-19 cases across 439 cities in 22 countries between 3 February 2020 and 31 August 2022. The full list of the cities included in the analysis is reported in Table S2; http://links.lww.com/EE/A311. The chosen cities represent an average of 26.4% of their country’s population, ranging from 5.5% in the Philippines to 100% in Kuwait (Table S3; http://links.lww.com/EE/A311). In total, the cities included in our study represent 4.7% of the world population and 16% of all reported cases. Figures 1 and 2 show the city locations and country-wide aggregated time series of daily reported COVID-19 cases per 100,000 inhabitants of each location. We can recognize the waves related to different variants as well as an incidence peak related to the Omicron variant in late 2021 and early 2022 for each country.

Table 1 shows the average, minimum, and maximum recorded exposures per country within the observation period. In Supplementary Figures S1-S5; http://links.lww.com/EE/A311, we present the daily country averages of the meteorological variables over the observation period. As expected, countries in tropical climates show less variation in the meteorological variables, especially mean temperature, RH, and AH. An overview of the governmental interventions against COVID-19 over time is given in the supplementary Figure S6; http://links.lww.com/EE/A311. Most countries started with stringent restrictions at the beginning of 2020, with a declining trend in stringency up to the summer of 2022. Figure S7; http://links.lww.com/EE/A311 shows the trend in vaccination coverage by country. In most countries, the vaccination coverage reaches a plateau at the end of 2021, with vaccination coverage over 60%, with the exceptions of Romania and South Africa, which have lower vaccination coverage (less than 40%).

**Table 1.** Summary of number of cities, observed COVID-19 cases, meteorological exposures, governmental stringency index, and vaccination coverage in the different countries

Country	Number of included cities	COVID-19 cases	Daily mean temperature Mean (range) [°C]	Daily mean RH Mean (range) [%]	Daily mean AH Mean (range) [g/m^3^]	Daily mean UV Mean (range) [W/m^3^]	Daily mean precipitation Mean (range) [mm]	Daily OXGRT SI Mean (range) (%)	Max vaccination coverage (%)
Australia	1	165,380	19.5 (10.5, 30.5)	73.4 (25.2, 98.1)	12.8 (3.8, 22.7)	186.8 (14.2, 366.6)	3.6 (0, 155.7)	54.6 (11.1, 78.2)	84.9
Brazil	18	465,1292	24.1 (4.2, 34.5)	74.2 (17.7, 99)	16.8 (3.7, 23.6)	209.9 (9.7, 381.1)	3.9 (0, 189.8)	56.1 (5.6, 81)	86.3
Canada	3	474,399	6.2 (−34.2, 30.9)	69.6 (27.3, 99)	6 (0.2, 16.9)	162.9 (2.6, 365.6)	3.3 (0, 91.1)	60.7 (2.8, 76.4)	85.4
Chile	3	286,659	12.9 (0.4, 23.9)	72.2 (21.4, 98.1)	8.2 (2.9, 14.3)	209.1 (3.9, 413)	1.9 (0, 59.9)	61.8 (25.7, 90.3)	92.0
Czech Republic	1	528,114	10.5 (−12.8, 26.9)	71.5 (33.4, 96.8)	7.5 (1.6, 16.1)	147.6 (6.7, 327.3)	2.2 (0, 83.4)	46.1 (11.1, 82.4)	66.4
Estonia	1	284,833	7.9 (−16.9, 26.8)	76.6 (39.9, 98.4)	6.9 (1.1, 16.7)	132 (1.9, 334.7)	2 (0, 39.9)	37.1 (0, 77.8)	65.3
Germany	11	4,470,963	11 (−12.8, 30.2)	73.6 (26.4, 99.2)	7.8 (1.5, 16.5)	142.5 (2.5, 339.5)	2.3 (0, 91.2)	53.4 (11.1, 85.2)	77.7
Italy	23	91,28185	15.9 (−5.3, 34.9)	69.9 (28.5, 98.6)	10 (1.5, 21.5)	191 (5.3, 348.3)	2.3 (0, 84.1)	60.3 (19.2, 93.5)	86.2
Japan	10	10,029,033	15.4 (−13.4, 31.2)	76.4 (36.5, 98.3)	11.6 (1.3, 24.8)	169.5 (10.3, 344.7)	4.7 (0, 184.8)	43.3 (19.4, 55.1)	84.0
Kuwait	1	713,588	27.8 (7.7, 41.9)	39.4 (14.5, 91.7)	10.2 (3.3, 26.2)	236.5 (27.2, 335.4)	0.2 (0, 29.4)	57.5 (5.6, 100)	80.6
Mexico	11	3,704,325	18.7 (−5.8, 34.6)	52.8 (3.2, 96.0)	8.5 (0.6, 19.4)	258.0 (28.7, 384.5)	1.8 (0, 106.2)	51.2 (0, 82.4)	75.3
The Netherlands	5	1,138,331	11.9 (−8.4, 28.7)	76.6 (34.1, 99)	8.5 (2.2, 18.1)	146.3 (3.5, 333.5)	2.3 (0, 38.3)	50.1 (5.6, 82.4)	72.7
Norway	1	197,687	7.9 (−15.8, 23.6)	75.2 (34.2, 99.9)	6.7 (1.1, 16.2)	129.1 (1, 340.2)	2.5 (0, 37.9)	41.1 (11.1, 79.6)	80.0
Peru	18	326,4185	15 (0.3, 30)	72.1 (9.8, 99.7)	10.3 (1.1, 23.5)	235.8 (34.8, 400.9)	3.6 (0, 106.1)	68.9 (13.9, 96.3)	87.3
The Philippines	4	505,631	27 (23.4, 31.9)	79.7 (56.2, 93.1)	20.9 (13.4, 24.2)	201.7 (21.5, 317.2)	6.7 (0, 186.7)	67.3 (25, 100)	66.2
Romania	8	1,367,363	11.9 (−13.2, 31.3)	69.4 (29.9, 99.4)	8 (1.2, 20.7)	163 (4.2, 338.2)	1.8 (0, 45.4)	52.2 (11.1, 87)	27.9
Singapore	1	1,865,432	27 (24.5, 28.9)	82.3 (65.9, 91.2)	21.7 (17, 23.6)	190.7 (26.9, 304)	7.3 (0, 90.8)	48.7 (25, 82.4)	91.0
South Africa	1	414,972	16.1 (9, 25.8)	74.7 (39.6, 95.2)	10.4 (5.6, 16.4)	202.8 (19, 396.6)	1.6 (0, 37.7)	52.3 (11.1, 88)	37.0
Spain	52	10,468,477	14.9 (−5.3, 35.3)	68.1 (9.3, 99.2)	8.9 (2.0, 22.2)	188.4 (5.5, 368.6)	1.8 (0, 77.0)	57.0 (0, 85.2)	86.5
Switzerland	8	2,400,454	10.6 (−9.7, 28.7)	73.6 (31.1, 99.6)	7.7 (1.4, 18.4)	164.7 (3.4, 352.2)	3.8 (0, 80.6)	41.7 (0, 73.2)	69.7
United Kingdom	54	7,425,396	10.7 (−4.8, 26.3)	79.3 (42.6, 99.4)	8.1 (2.5, 16.9)	130.4 (1.2, 351.2)	2.4 (0, 48.3)	53.2 (11.1, 88)	79.4
United States	204	32,576,603	14.4 (−30.2, 40.4)	66.7 (3.1, 99.9)	9.5 (0.3, 25.3)	188.8 (2.2, 385.1)	2.9 (0, 246)	57.3 (0, 75.5)	77.8

### Association between COVID-19 cases and meteorological variables

**Figure 3.**
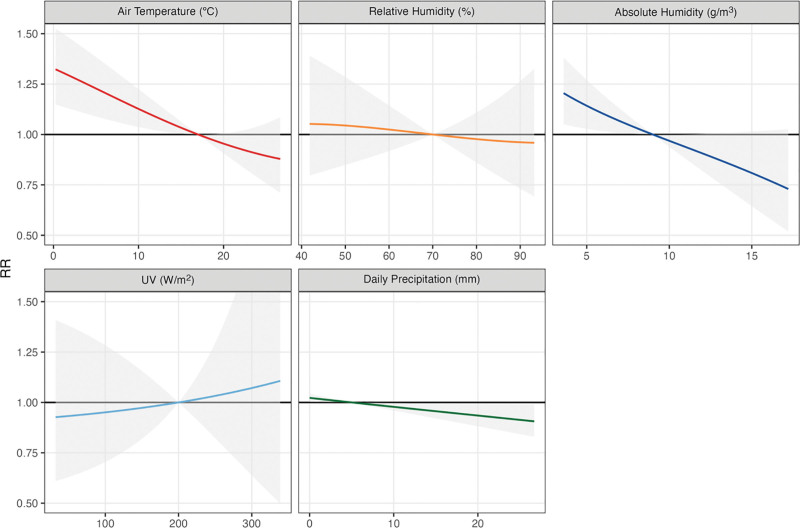
Pooled association curves between COVID-19 cases and meteorological variables, with a lag of up to 14 days.

Figure 3 shows the pooled association curves, representing overall results across all cities from the meta-analysis models for meteorological variables considered independently. Low temperatures were associated with a higher risk of infection. At 5 °C, the risk of COVID-19 incidence is 1.22-fold higher (95% CI = 1.09, 1.38) compared to a reference level at 17.0 °C. The exposure lag association indicated increased RRs with a 3-day lag after temperature exposure, peaked at 6–8 days, and decayed by the end of the observed 13-day lag period (Figure S8; http://links.lww.com/EE/A311). We observed a substantial heterogeneity in the meta-analytic model (I^2^ = 78.4%). Figure 3 indicates that AH had an inverse association similar to temperature. Compared with the median value of 9.0 g/m³ there was a 1.14-fold increased RR at the AH of 5.0 g/m³ (95% CI = 1.03, 1.27). The RRs increased (RR >1.00) between 2 and 9 days of lag (Figure S8; http://links.lww.com/EE/A311). We did not observe an association for RH and solar UV radiation but observed a tendency of increased risk on days without precipitation.

As shown in Supplementary Figures S9–S12; http://links.lww.com/EE/A311, when we considered models with temperature and each of the other meteorological variables, temperature, and precipitation seem to be independent predictors, whereas the association between AH and COVID-19 incidence is reduced when temperature is included in the model. Table S9; http://links.lww.com/EE/A311 presents a correlation matrix between all the meteorological variables, which confirms that the strongest correlation is between AH and temperature.

The results from the sensitivity analysis that consider a linear relationship (Table S4 and Figure S13; http://links.lww.com/EE/A311) show similar results from our main model, but with a stronger inverse association between RH and COVID-19 incidence. The lagged effects of the linear relationship showed an incidence peak at 6-8 days after temperature exposure, similarily to the main model (Figure S14; http://links.lww.com/EE/A311). The seasonality analysis showed that the effect was stronger during winter months for temperature and AH (Figure S17; http://links.lww.com/EE/A311).

### Temporal variation of the association between COVID-19 cases and meteorological variables

#### Vaccination coverage

We observed some inconsistent patterns in the analysis of the effect of meteorological variables on COVID-19 spread in periods with different vaccination coverage.

**Figure 4.**
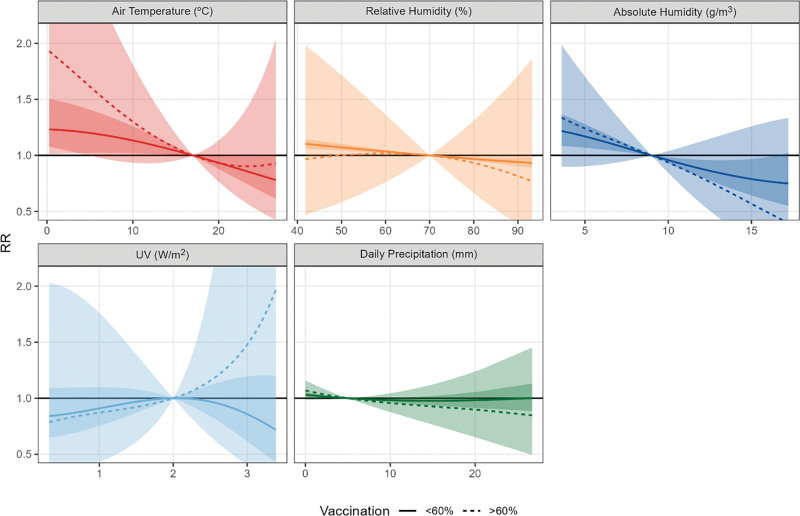
Pooled association curves between COVID-19 cases and meteorological variables by periods with low (<60%) and high (>60%) vaccination coverage.

For average temperature as exposure, the model with interaction shows higher RRs and uncertainty in the period with higher coverage (Table 2 and Figure 4). We see a similar tendency for the RRs for AH. RH shows a very slight opposite tendency, with inverse relationships in the period with low vaccination coverage and no association if the vaccination coverage was above 60%. UV shows a consistent pattern of no association in the two periods with different vaccination coverage, while precipitation shows consistent inverse associations, with higher RRs in days without precipitation.

The higher uncertainty in the periods with a higher vaccination rate seems unexplained by lower power as there was a higher number of cases (56,990,369) compared to the period with lower vaccination rates (n = 39,070,933).

Our sensitivity analysis revealed that the modifier effect of the vaccination period on the association between temperature and COVID-19 incidence was stronger when using the definition of fully vaccinated people. (Table S6; http://links.lww.com/EE/A311). Although there is a lot of uncertainty, the RR in the >60% vaccination period was higher (RR: 2.37; CI = 1.19, 4.75) compared to the lower vaccination period (RR: 1.10; 95% CI = 0.91, 1.33). When considering the linear relationship, the association for temperature, AH, and RH were both stronger during the <60% vaccination coverage period (Table S7 and Figure S15; http://links.lww.com/EE/A311).

**Table 2.** Associations (RRs and 95% CI) between meteorological variables and COVID-19 incidence, by total RR and vaccination coverage threshold of 60%

	RRs (n cases = 96,061,302)	I^2^	Vaccination coverage <60% (n cases = 39,070,933)	Vaccination coverage >60% (n cases = 56,990,369)
Mean temperature (5 °C vs. 17 °C)	1.22 (1.09, 1.38)	78.4	1.20 (1.02, 1.40)	1.60 (0.99, 2.57)
RH (60% vs. 70%)	1.02 (0.92, 1.14)	75.5	1.03 (1.02, 1.05)	1.02 (0.77, 1.35)
AH (5 g/m^3^ vs. 9 g/m^3^)	1.14 (1.03, 1.27)	73.7	1.17 (1.07, 1.28)	1.23 (0.89, 1.69)
UV (100 W/m^2^ vs. 200 W/m^2^)	0.95 (0.71, 1.28)	77.8	0.91 (0.76, 1.10)	0.87 (0.44, 1.74)
Precipitation (0 mm vs. 5 mm)	1.02 (1.00, 1.04)	63.0	1.03 (1.01, 1.05)	1.05 (0.97, 1.15)

Relative risks are for a chosen value compared to a reference value, for example, the overall risk at a temperature of 5 °C is 1.22 times higher compared to the risk at 17 °C.

#### Strains

**Figure 5.**
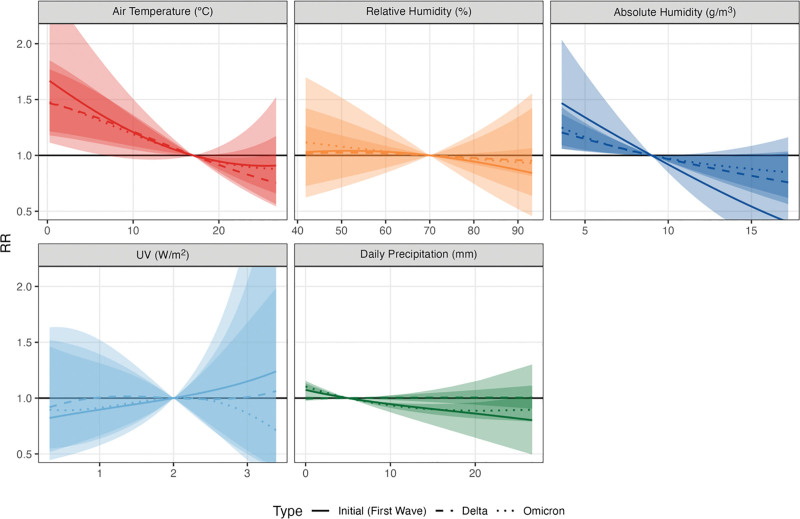
Pooled association curves between COVID-19 cases and meteorological variables by periods with different dominant strains [Initial (First wave), Delta, Omicron].

Our analyses of associations between meteorological variables and COVID-19 incidence by time periods with different dominant strains show a relatively consistent pattern of RR across periods. Temperature, AH, and precipitation show consistent associations with COVID-19 incidence during the observation period (Table 3 and Figure 5). In contrast, we see no association in the three periods per RH and UV. In the sensitivity, considering a linear relationship, we did however find an inverse association for UV during the Omicron period (Table S8 and Figure S16; http://links.lww.com/EE/A311).

**Table 3.** Associations (RRs and 95% CI) between meteorological variables and COVID-19 incidence, by time periods with different dominant strains

	Initial (first wave) (n cases = 25,702,294)	Delta (n cases = 14,510,657)	Omicron (n cases = 48,085,841)
Mean temperature (5 °C vs. 17 °C)	1.41 (1.02, 1.95)	1.35 (1.15, 1.59)	1.34 (1.12, 1.61)
RH (60% vs. 70%)	1.03 (0.85, 1.26)	1.02 (0.83, 1.16)	1.04 (0.99, 1.08)
AH (5 g/m^3^ vs. 9 g/m^3^)	1.34 (1.03, 1.74)	1.15 (1.04, 1.26)	1.16 (1.05, 1.29)
UV (100 W/m^2^ vs. 200 W/m^2^)	0.90 (0.58, 1.39)	1.01 (0.67, 1.52)	0.91 (0.64, 1.31)
Precipitation (0 mm vs. 5 mm)	1.07 (1.00, 1.15)	1.00 (0.98, 1.20)	1.10 (1.08, 1.13)

Relative risks are for a chosen value compared to a reference value, for example, the risk at a temperature of 5 °C is 1.41 times higher compared to the risk at 17 °C during the Initial wave.

## Discussion

We found evidence of an association between COVID-19 incidence and temperature. At temperatures of 5 °C, the risk of COVID-19 incidence is 1.22 times higher compared with a reference level of 17 °C, with the exposure lag association reaching a peak at 6–8 days. Additionally, we observed an inverse association for AH, with a 1.13-fold increased risk at 5.0 g/m³ compared with the median value of 9.0 g/m³, though this appeared to be driven by its correlation with temperature (Table S9; http://links.lww.com/EE/A311). We also observed a tendency of increased risk on days without precipitation and no association for RH and solar radiation. When we analyzed these factors for dominant strain and vaccination levels, we found little evidence for interaction with meteorological factors.

This study is a continuing analysis of a preceding study conducted using data from the first wave of COVID-19. Our findings of an association between mean temperature and AH with COVID-19 confirmed the results from the previous analysis. Days with lower temperature and lower AH are associated with higher COVID-19 incidence.^10^ We found no evidence for associations with RH or UV; other studies have shown similar results.^4,6,11,12^

Since the first COVID-19 cases emerged and spread globally, many articles have been published on meteorological factors and their association with COVID-19. Previous studies have shown more inconclusive results, but they tended to only cover a short timeframe. Baker et al^8^ hypothesized that at the beginning of an outbreak, meteorological factors only play a minor role in transmission. However, when an outbreak stabilizes, there might be a clearer association. Much research on meteorological factors and COVID-19 was done using different methodologies. A systematic review^25^ of studies published within the first 2 years of the pandemic found 289 relevant papers. Over a third of those articles did not consider lag effects in their analysis. On the other hand, we considered a lag of up to 14 days for each meteorological variable. Furthermore, our analysis included data covering 2.5 years, which is much longer than the average observation time of 123 days, as reported by Tan et al.^25^

Our results strengthen the evidence of previous studies on the association between temperature and COVID-19. Wang et al^26^ found that increased temperature was associated with a lower transmission. Interestingly, they also found a negative correlation between high RH and COVID-19 transmission. In our study, we found a negative correlation between high AH, though this was explained by its correlation with temperature; moreover, we found no correlation for RH. On the other hand, they did find a small positive association with increased UV exposure, while our results were not statistically significant.

Previous modeling studies showed that the effect of meteorological variables should be stronger in an immunized population.^8^ Our study included vaccination rates and dominant strains as possible effect modifiers. The result of this analysis, however, does not fully support the hypothesis for a higher impact of meteorological variables on immunized persons, as the associations for both temperature and AH were very uncertain in the high-vaccination period. One limitation was that we used country-wide vaccination rates, which might not be fully representative of the vaccination rates for the included cities. We did use state-specific vaccination rates for the United States to account for the large variability in vaccination coverage between the different states; however, we did not use any vaccination rates on a smaller spatial scale for the other countries, which might experience some level of variability as well. While research has shown that vaccination rates are strongly associated with a reduction in COVID-19 incidence, research on vaccination rates as an effect modifier of environmental impacts on COVID-19 is still lacking. The difference in the relationship between temperature and specific humidity, respectively, and COVID-19 transmission among populations with different immunization levels was investigated by Villatoro-Garcia et al.^27^ They found that COVID-19 transmission fell slightly during periods with higher temperatures and higher humidity in vaccinated populations. On the other hand, Hasan et al^28^ found that when vaccination rates were included in the model, there was no significant change in the impact of meteorological factors.

Similarly, little research has examined different strains as effect modifiers. We found no differences in meteorological impacts in periods with different strains. To our best knowledge, this is the first study to investigate the association between the different strains and the impact of meteorological factors on COVID-19 transmission. Looking at other environmental factors, a study by Ma et al^29^ found that in later strains, such as Delta and Omicron, susceptibility to COVID-19 significantly increased with increased exposure to air pollution.

This study adds to the literature on how COVID-19 incidence is impacted by meteorological variables, which can give us insights on where to look for mechanisms impacting the incidence. We specifically considered temperature, AH, RH, precipitation, and solar radiation as the meteorological factors. Some mechanistic studies undertaken in the laboratory found that the stability and viability of viral particles is higher in lower temperatures, which would explain why in our analysis we found lower temperatures to have higher COVID-19 transmission.^30,31^ According to animal experiments, lower temperature, and therefore lower blood circulation, could impact the body’s ability to fight off respiratory viruses due to impaired adaptive immunity.^32,33^ However, much of the environmental impact can also be explained by the related human behavior. During winter months, people tend to stay indoors in heated environments and closed spaces, resulting in human interaction in smaller spaces, which can increase spread. Public transport might be more crowded during days with lower temperatures, as people are less likely to commute by foot or bicycle in colder weather. Being surrounded by more people in a smaller space increases the risk of being exposed to someone who is infected with COVID-19.^34,35^

We found that lower AH is associated with a higher risk of COVID-19 infection. This positive association is explainable by the theory that droplets containing viruses evaporate in dry conditions. Through the evaporation, dry nuclei would be formed, which could float in the air for longer times.^36^ However, we found no evidence for an effect of AH after controlling for temperature.

While we found no evidence for the UV-COVID-19 association, the general trend seemed to be that lower UV exposure is associated with higher transmission, though the association reversed when controlled for temperature. Studies showed that UV inactivates viruses in both the air and on surfaces. Therefore, higher UV could decrease the exposure to SARS-CoV-19.^37^ Comparable to temperature, the relationship between UV and COVID-19 incidence could also be explained by human behavior, as people tend to go outside in sunny weather and spread out more, instead of being in closer proximity indoors. Similar to our study, Balboni et al^38^. found a negative association between higher temperature and COVID-19 spread but no significant association with RH.^38^

The sensitivity analysis only showed a small difference in results when considering different vaccination thresholds or definition of fully vaccinated. When considering linear associations, we found slightly stronger evidence of associations, and when analyzing the effect modification of vaccination rates, we also found a stronger association in the low-vaccination periods (Table S4–S7; http://links.lww.com/EE/A311). We considered seasonality in our sensitivity analysis and found that the RR is mainly present during winter and statistically significant for temperature and AH (Figure S17; http://links.lww.com/EE/A311). One explanation could be population behavior, as people have the tendency to stay indoors in more crowded areas during low temperatures in winter but tend to spend more time outdoors and spread out when the temperature is higher. Another mechanism influencing this association could be that particles stay in the air longer under conditions of high levels of AH compared to levels of low AH.

A key strength of our study was the size, breadth, and spatiotemporal granularity of the data we used. In total, we collected data for 96.1 million COVID-19 cases which represent 16% of the total cases (607,913,476) registered by 31 August 2022, according to Worldometer.^39^ First, the data included daily COVID-19 cases for 439 cities in 22 countries spanning diverse settings across the globe, making it quite representative, and by selecting all the cities that are in the MCC network we avoided a selection bias. Cities included in our study represent 4.7% of the world population, which gives the analysis a lot of power and reduction of biases in the results. The cities in the MCC network also tend to be the largest cities in their respective countries and represent an average of 26.4% of their country’s population. Second, the observation period was much longer compared with previous studies on COVID-19. Using data spanning 2.5 years, we were able to analyze several incidence peaks, over different periods with varying vaccination coverage and dominant strains. To our knowledge, our study included more locations, analyzed as smaller spatial units, and longer observation periods than most published literature on the subject.^4,25^ Several ecological and time-varying confounders were considered and incorporated in the analysis.

The study also has a few possible shortcomings. While the study area is global, certain regions and locations are still underrepresented, as the cities included are not distributed proportionally over the human population. Furthermore, definitions of COVID-19 cases and reporting practices vary from country to country, and the GSI, measured at country level, could not fully capture governmental interventions as local interventions might vary throughout a country and impact the efficiency of governmental interventions.^40^

Strengthening previous research, this study shows evidence of the relationship between lower temperatures and AH levels and COVID-19 incidence. We did not find a significant modifier effect of vaccination coverage on the associations between meteorological factors and COVID-19. We also found no clear differences in the associations between the dominant strains. This is one of the first empirical studies looking at the modifying effect of vaccination coverage and dominant strains on the meteorology-COVID-19 association.

## ACKNOWLEDGEMENTS

We acknowledge the contributions and support of all authors for this paper.

## Supplementary Material
