# Supplementary material for: Meteorological factors, population immunity, and COVID-19 incidence: A global multi-city analysis
Source: Environ Epidemiol. 2024 Nov 11;8(6):e338. doi: 10.1097/EE9.0000000000000338 (PMC11557119; doi:10.1097/EE9.0000000000000338)
Supplement: Supplementary file 1 [file ee9-8-e338-s001.pdf]

Figure S1. Daily mean average temperature time-series aggregated for all cities by country 2020-2022.

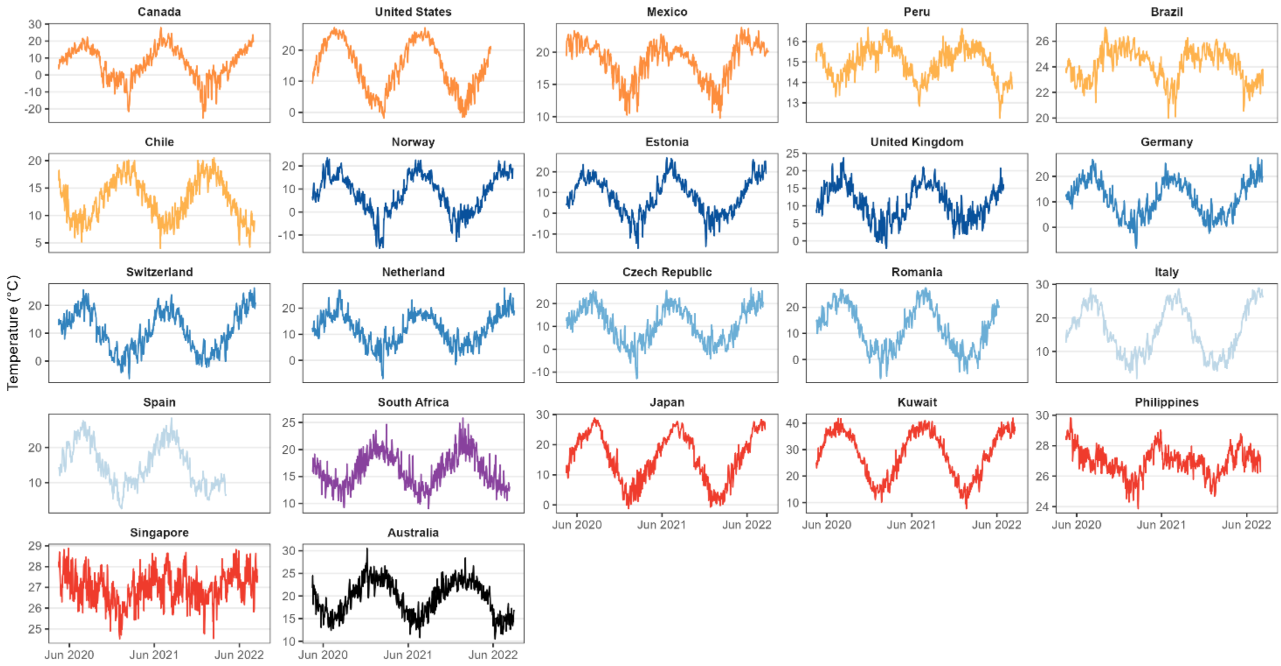

Figure S2. Daily mean exposure RH time-series aggregated for all cities by country 2020-2022.

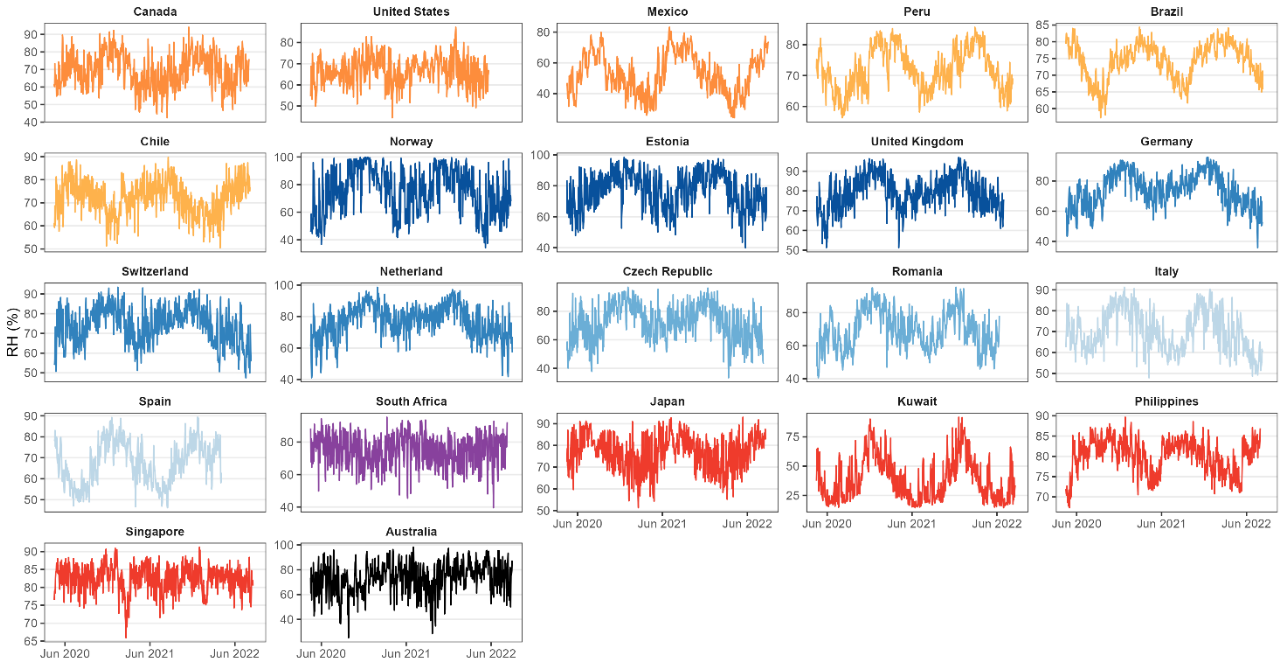

Figure S3. Daily mean exposure AH time-series aggregated for all cities by country 2020-2022.

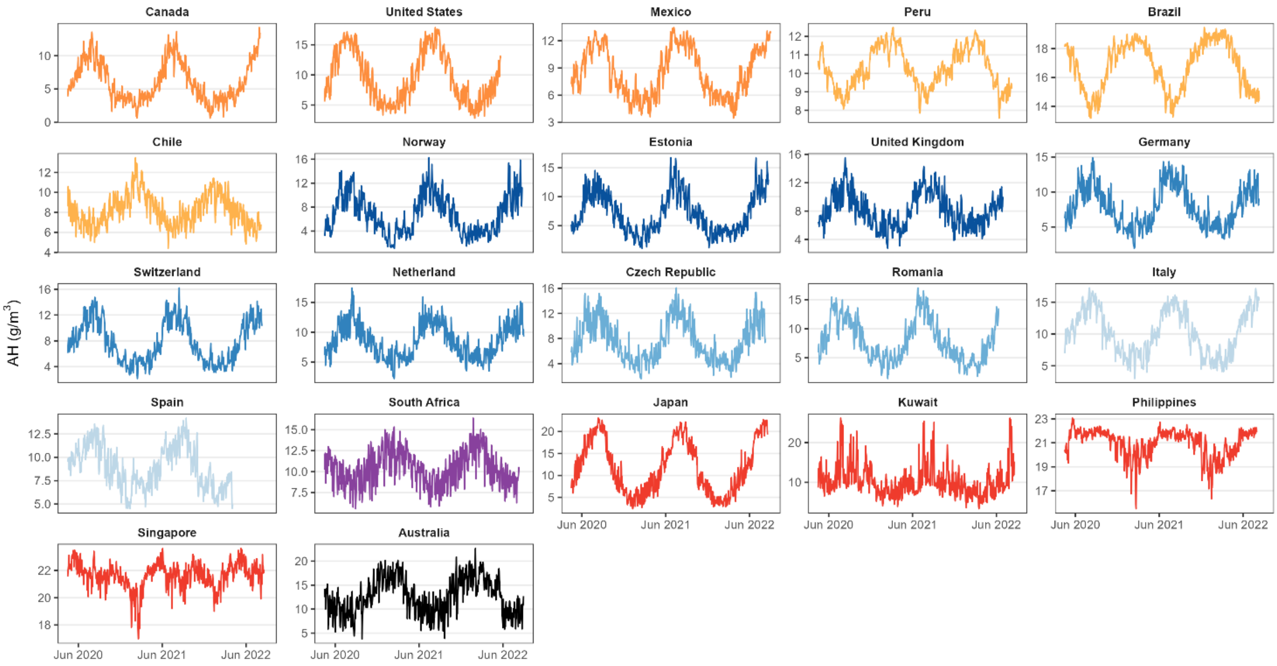

Figure S4. Daily mean exposure UV time-series aggregated for all cities by country 2020-2022.

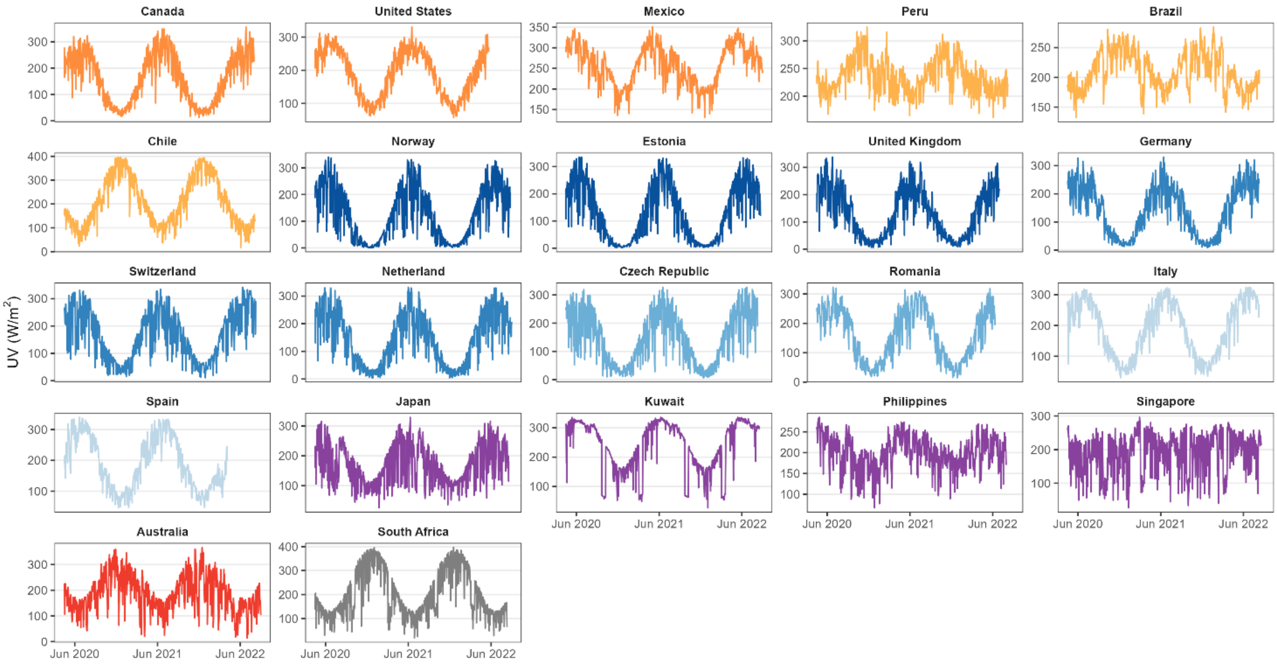

Figure S5. Daily mean exposure precipitation time-series aggregated for all cities by country 2020-2022.

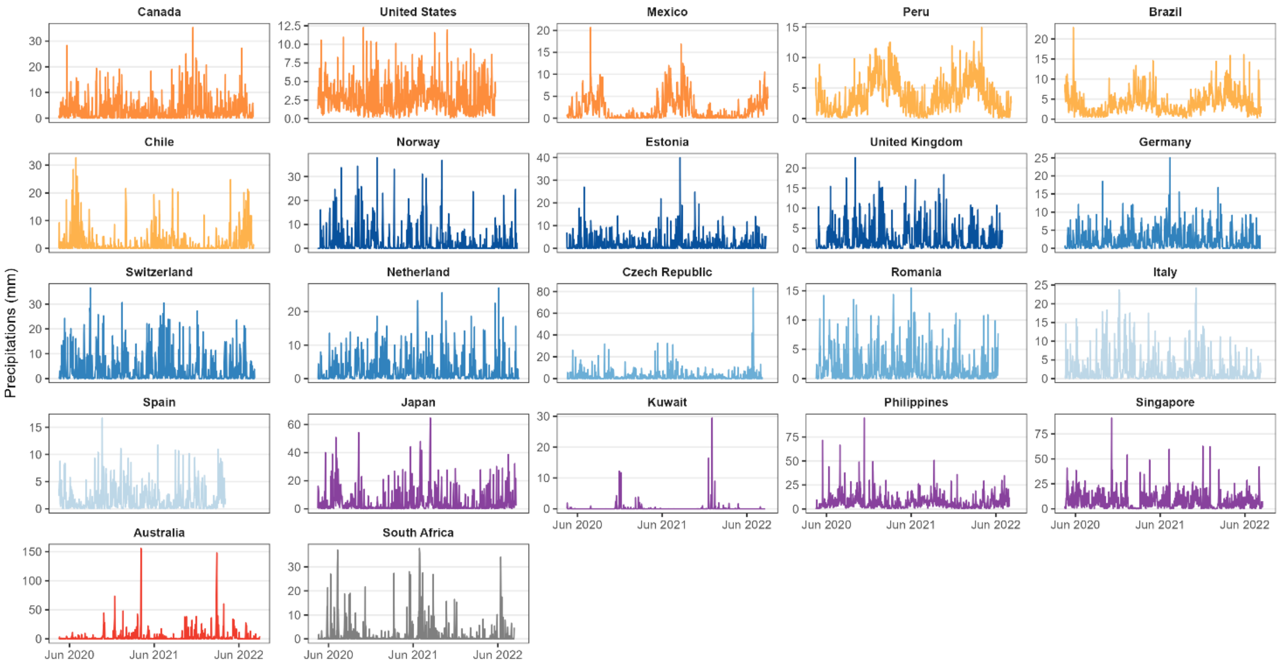

Figure S6. Daily mean GSI time-series aggregated for all cities by country 2020-2022

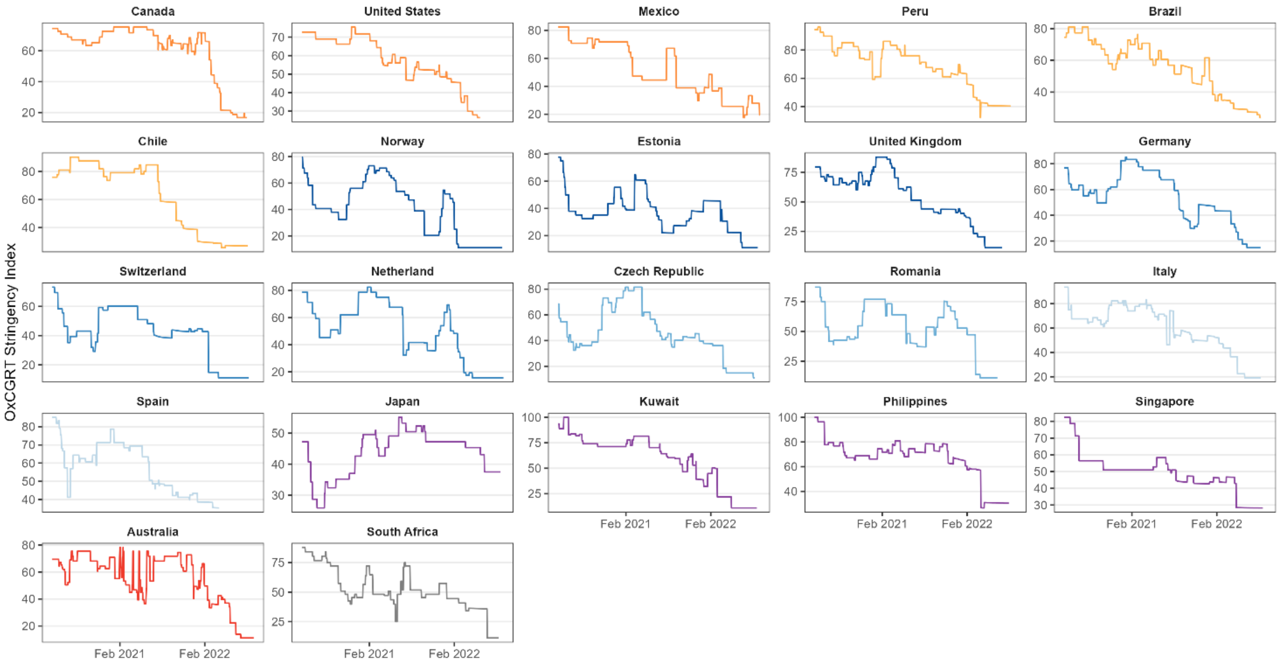

Figure S7. Daily mean vaccination coverage time-series aggregated by all cities per country 2020-2022.

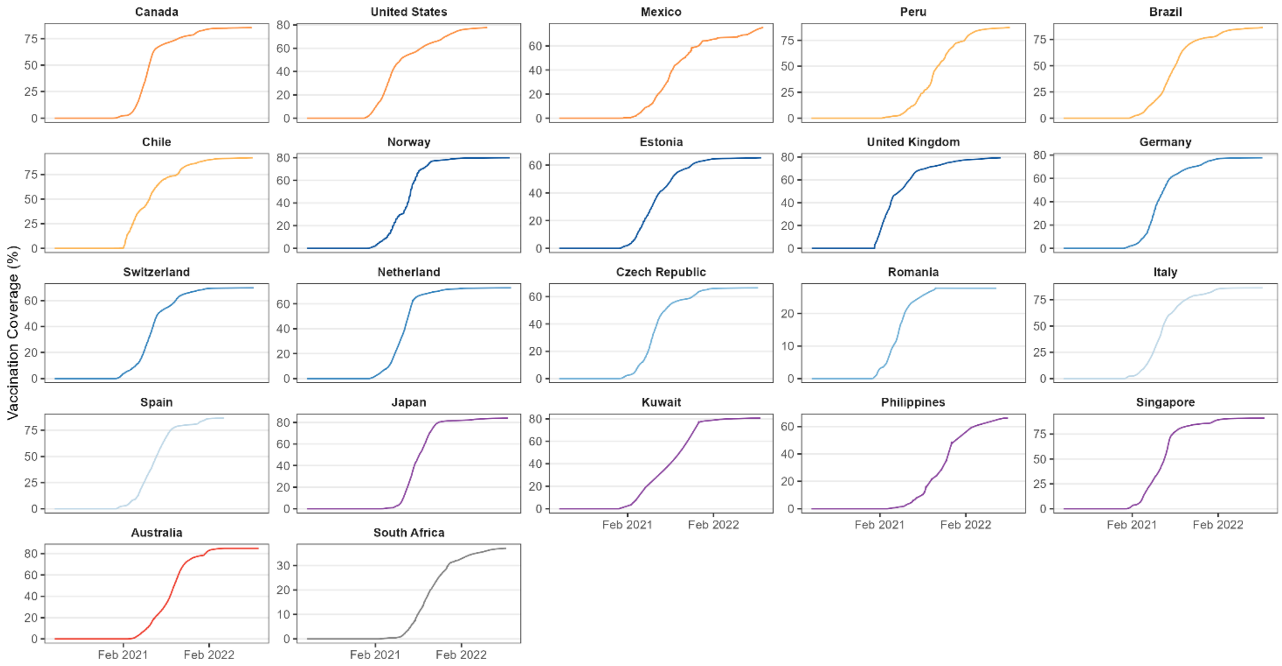

Figure S8. Association between COVID-19 cases and meteorological variables (Lagged effects).

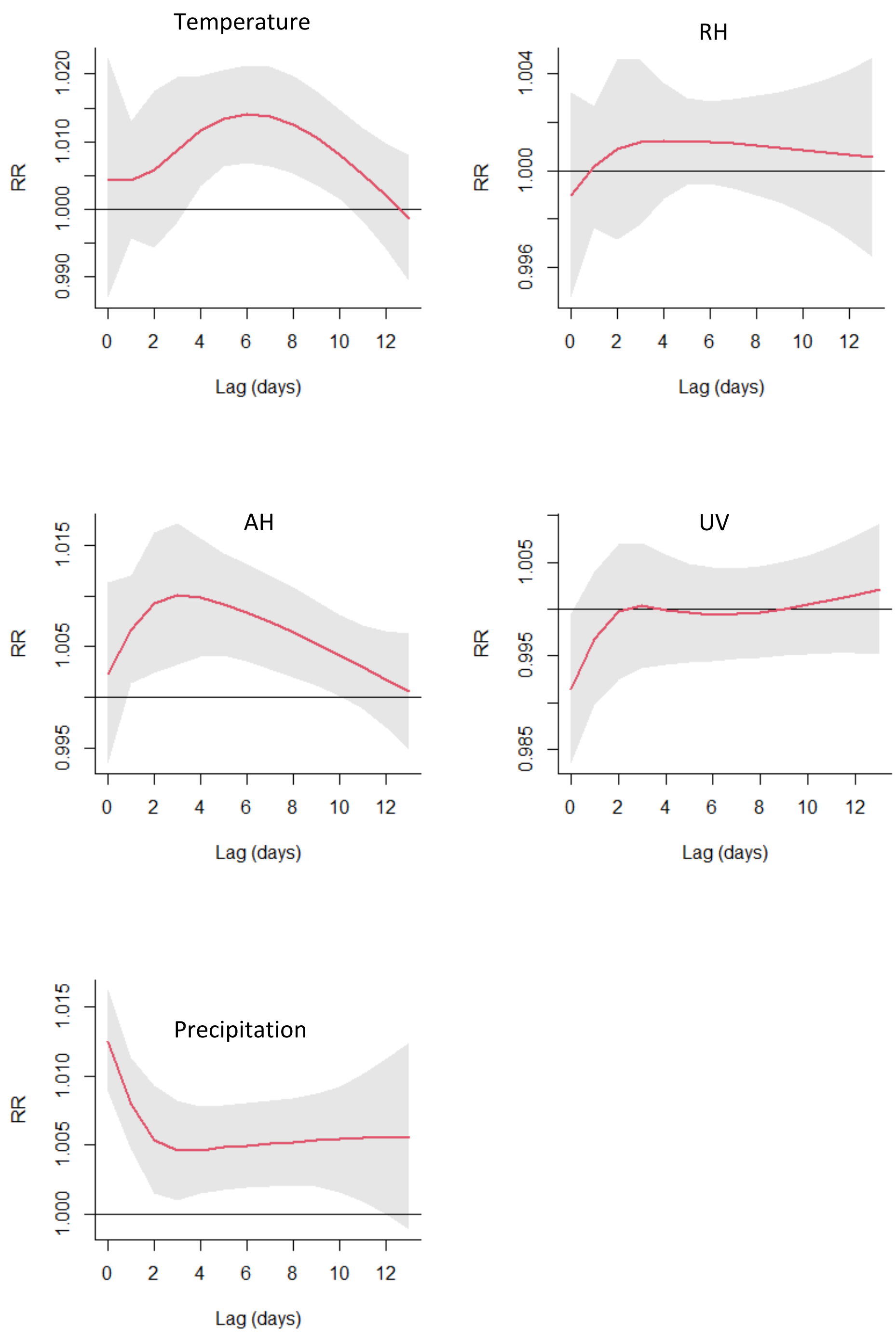

Figure S9. Association between COVID-19 cases with temperature and RH (Multivariable Model).

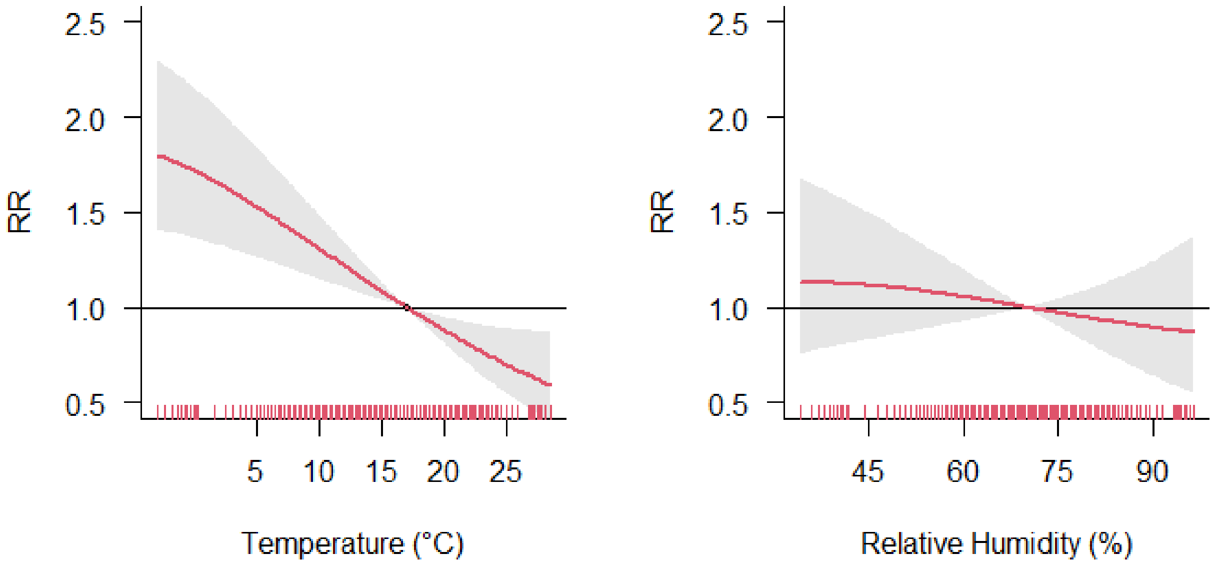

Figure S10. Association between COVID-19 cases with temperature and AH (Multivariable Model).

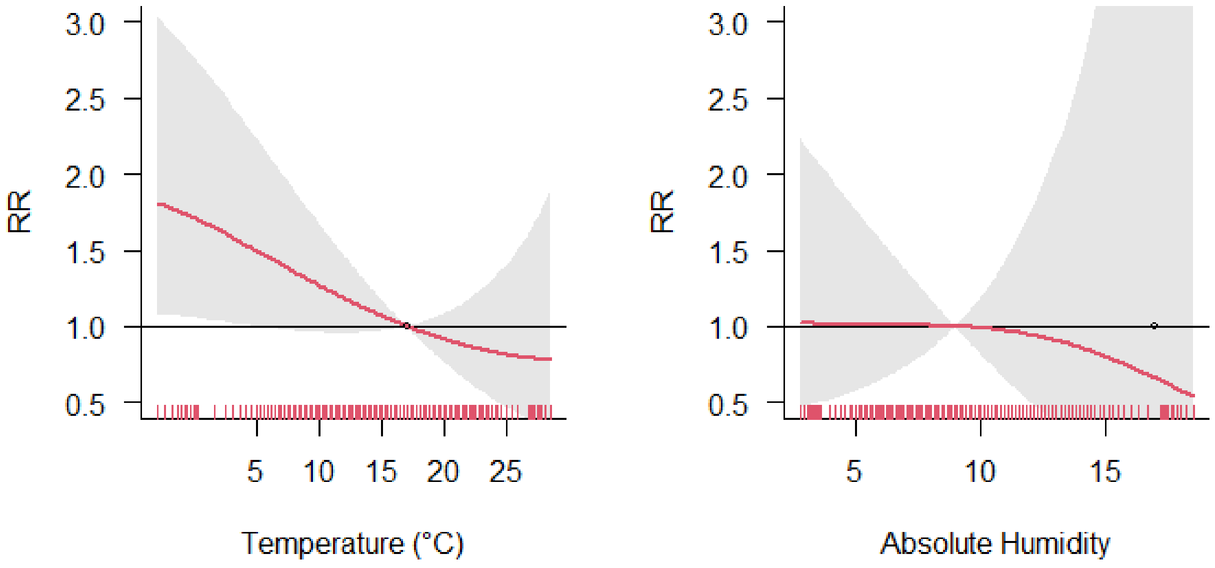

Figure S11. Association between COVID-19 cases with temperature and UV (Multivariable Model).

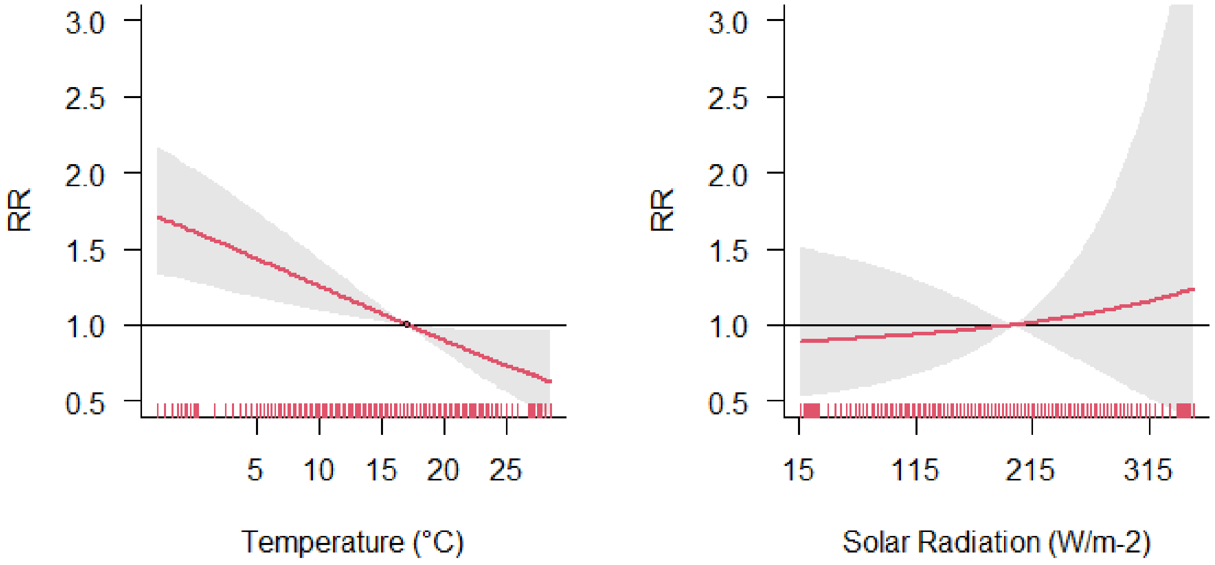

Figure S12. Association between COVID-19 cases with temperature and precipitation (Multivariable Model).

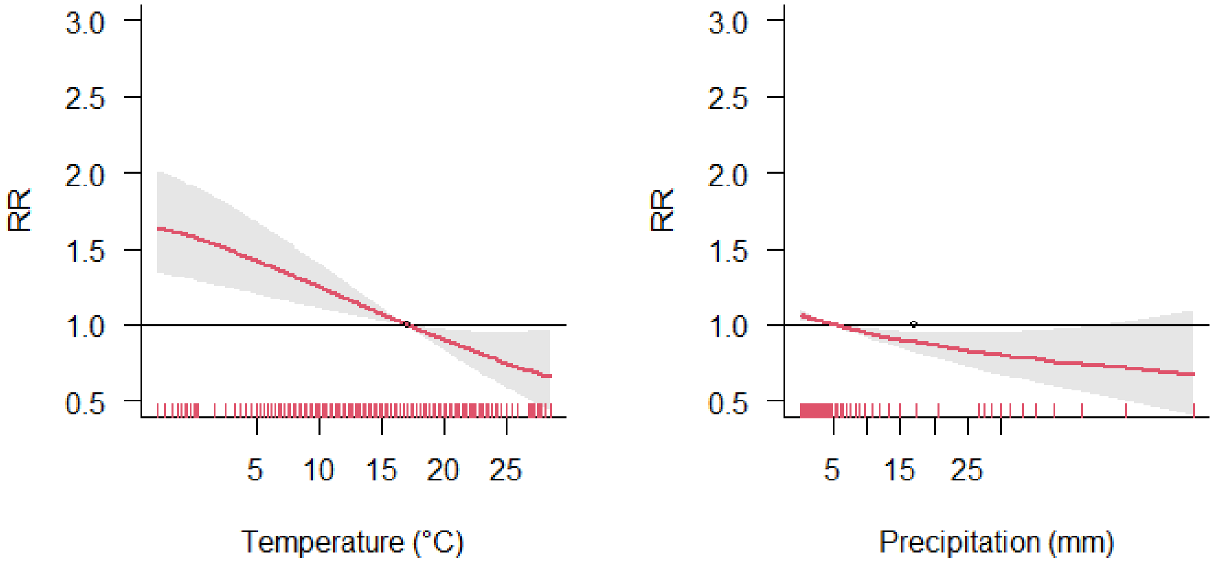

Figure S13: Sensitivity Analysis: Linear relationship

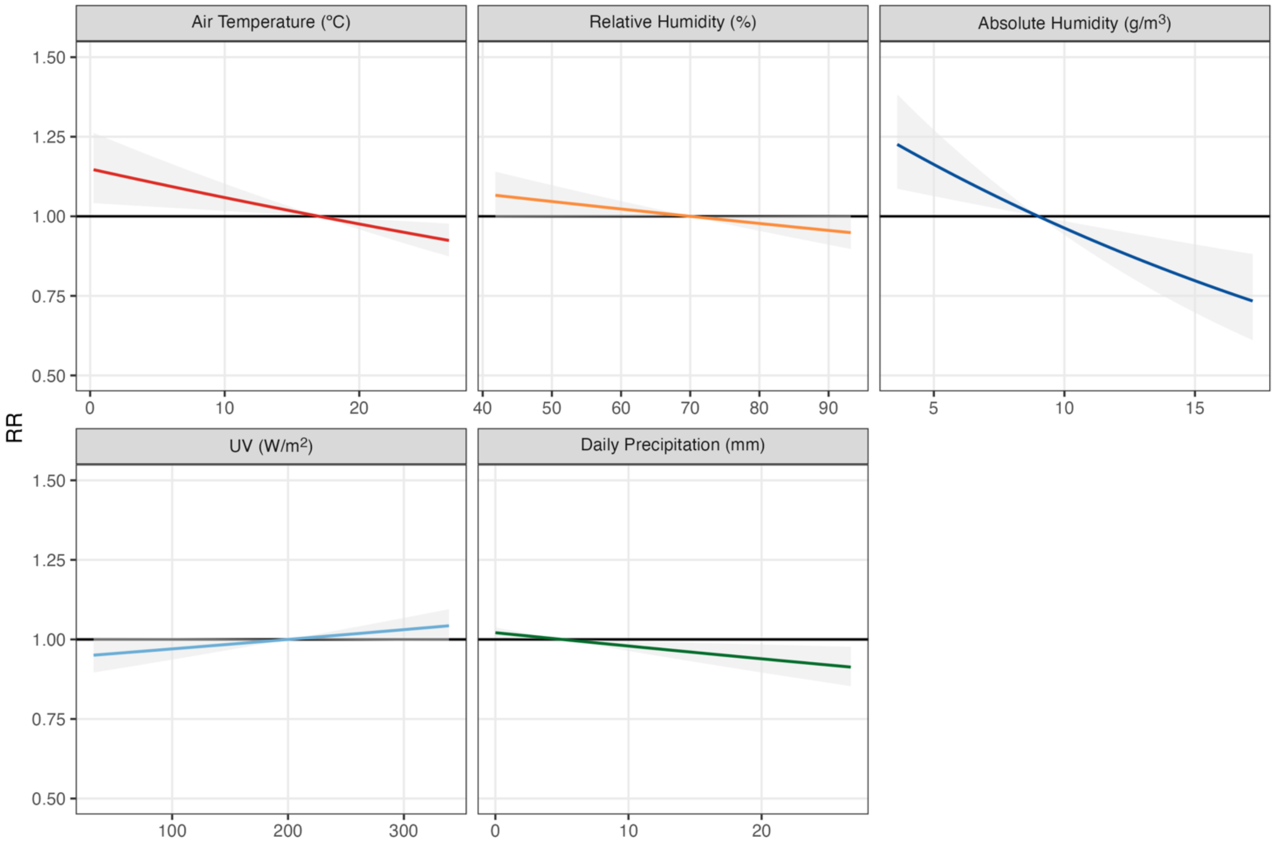

Figure S14: Sensitivity Analysis: Lag effects of linear relationship

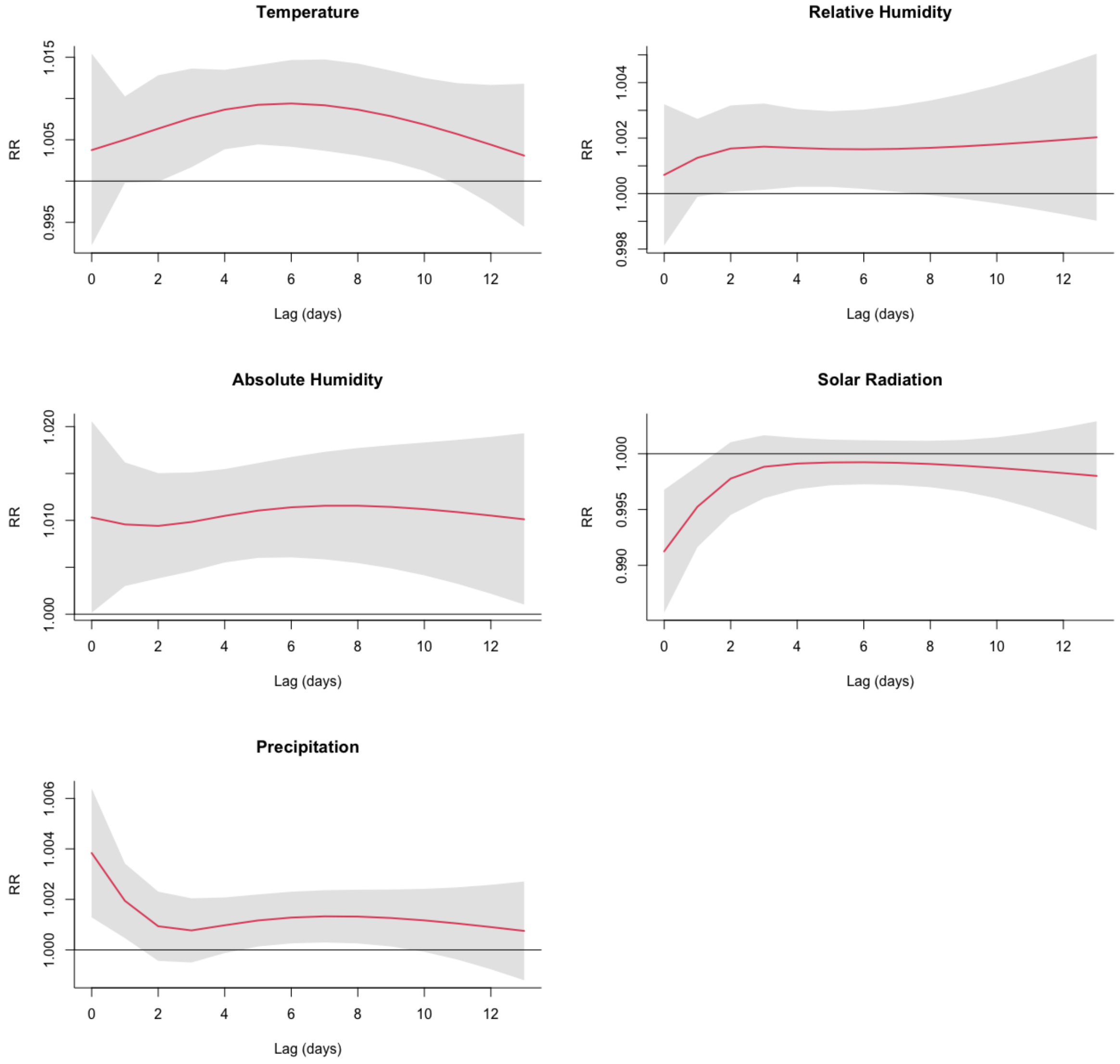

Figure S15: Sensitivity Analysis: Linear relationship among different vaccination coverages

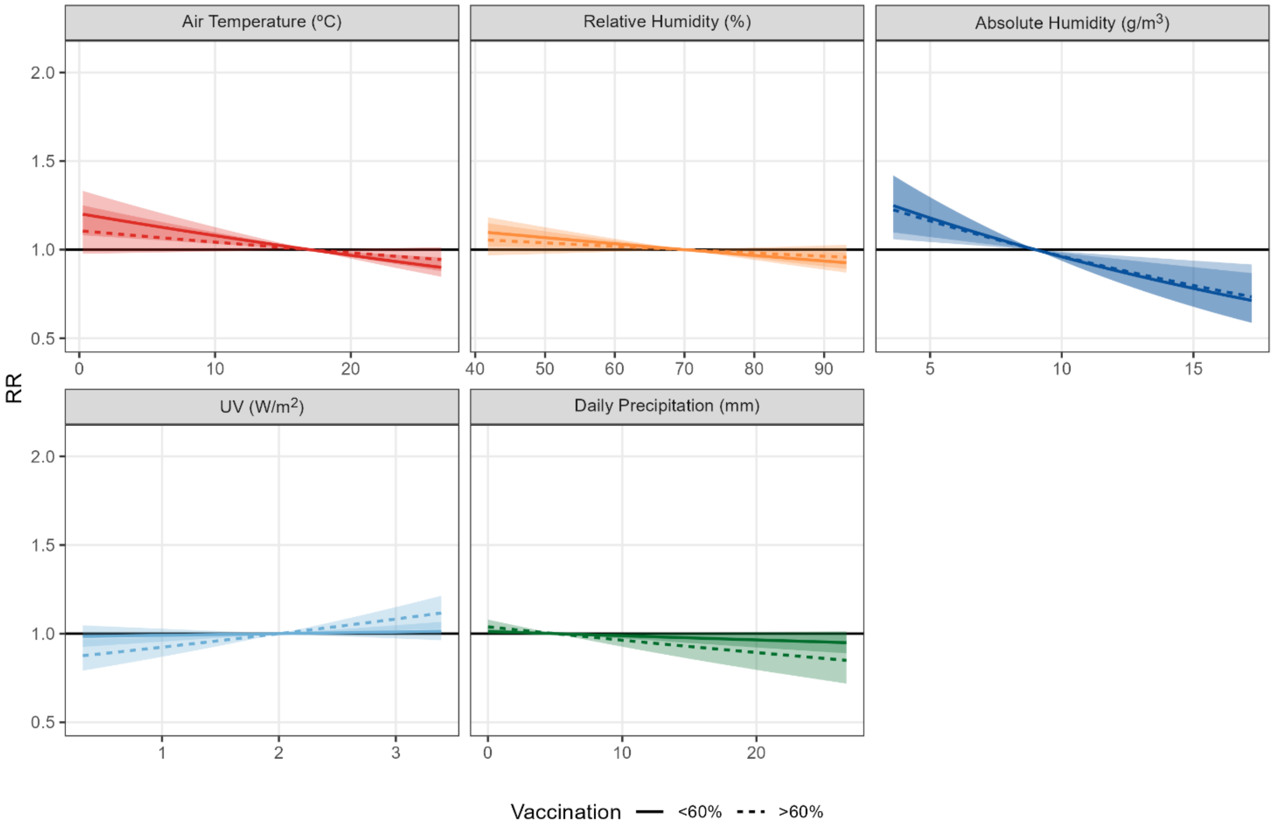

Figure S16: Sensitivity Analysis: Linear relationship among different dominant strains

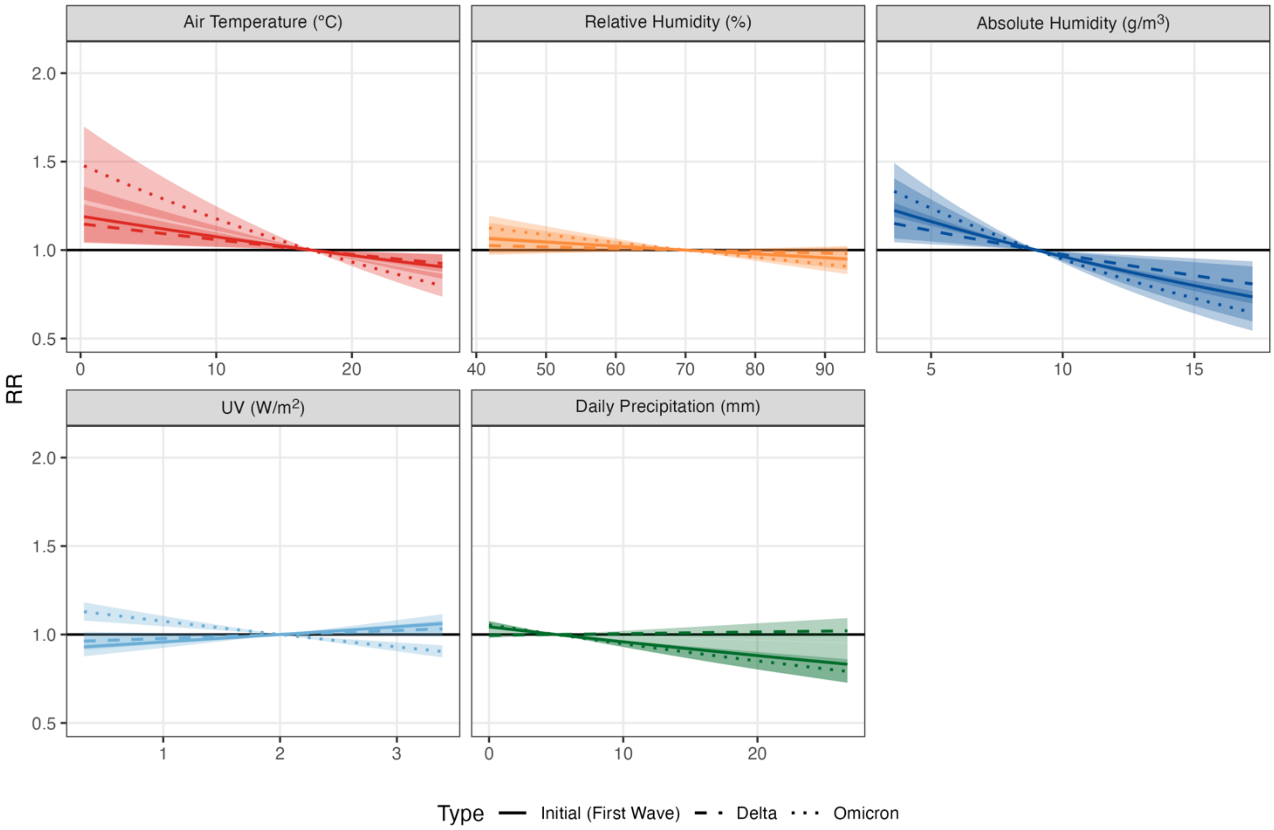

Figure S17: Seasonal associations of COVID-19 transmission and meteorological variables

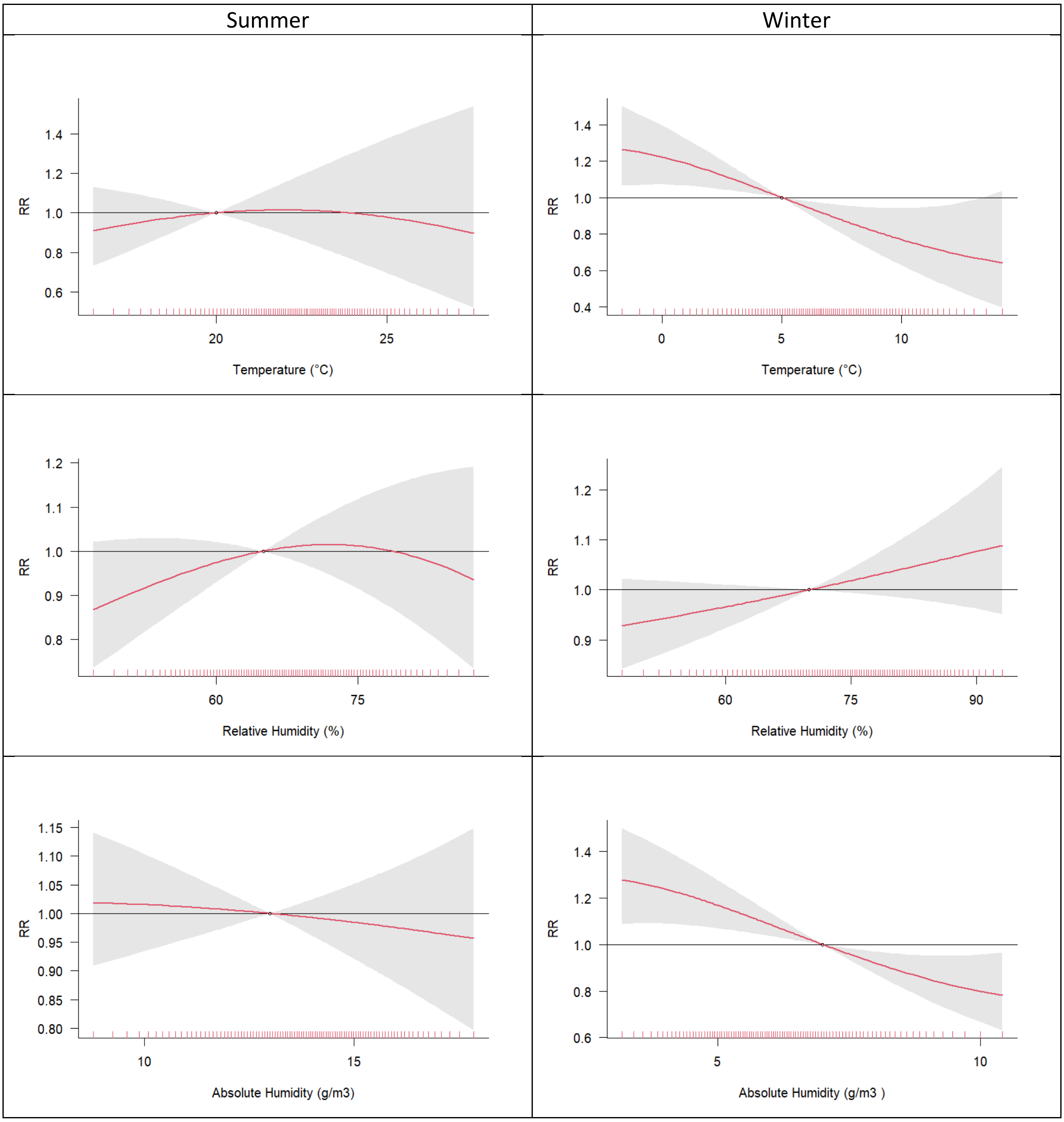

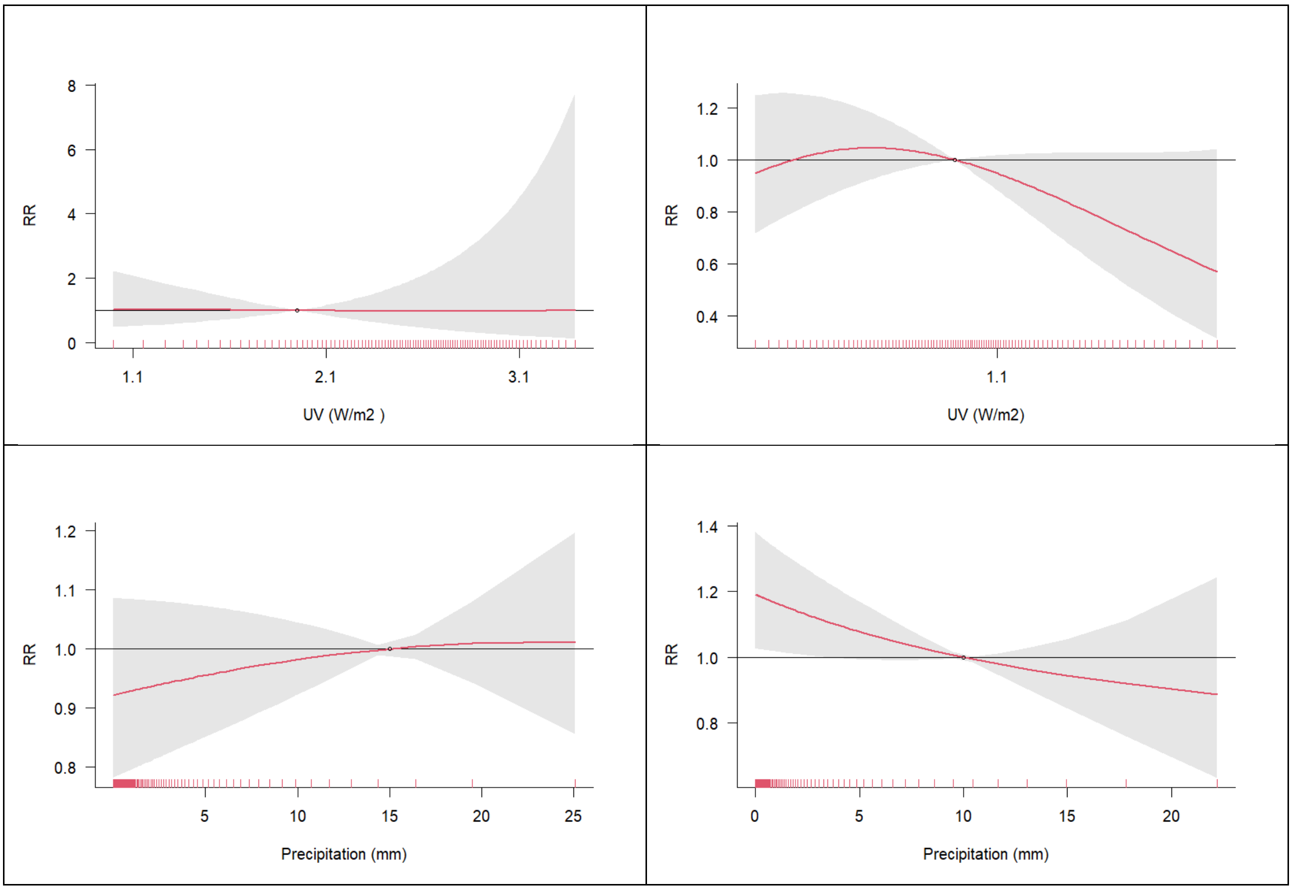

RR
8
6
4
2
0
1.1
2.1
3.1
UV (W/m2)
RR
1.2
1.0
0.8
0.6
0.4
1.1
UV (W/m2)
RR
1.2
1.1
1.0
0.9
0.8
5
10
15
20
25
Precipitation (mm)
RR
1.4
1.2
1.0
0.8
0
5
10
15
20
Precipitation (mm)

Table S1. COVID-19 data sources

**Canada**	Sources
Alberta	https://www.alberta.ca/data/stats/covid-19-alberta-statistics-data.csv
British Columbia	http://www.bccdc.ca/Health-Info-Site/Documents/BCCDC_COVID19_Dashboard_Case_Details.csv
Manitoba	https://www.inspq.qc.ca/covid-19/donnees
Montreal	https://www.inspq.qc.ca/covid-19/donnees
Ontario	https://data.ontario.ca/dataset/f4112442-bdc8-45d2-be3c-12efae72fb27/resource/455fd63b-603d-4608-8216-7d8647f43350/download/conposcovidloc.csv
Saskatchewan	https://dashboard.saskatchewan.ca/health-wellness/covid-19/cases
**Mexico**	https://www.arcgis.com/sharing/rest/content/items/f10774f1c63e40168479a1feb6c7ca74/data
**United States**	https://data.cdc.gov/Case-Surveillance/COVID-19-Case-Surveillance-Restricted-Access-Detai/mbd7-r32t/about_data
**Brazil**	https://transparencia.registrocivil.org.br/painel-registral/especial-covid
**Chile**	Ministry of Science, Technology, Knowledge, and Innovation (Minciencia): URL https://raw.githubusercontent.com/MinCiencia/Datos-COVID19/master/output/producto1/Covid-19.csv
**Peru**	Ministry of Health (MINSA): URL https://www.datosabiertos.gob.pe/dataset/casos-positivos-por-covid-19-ministerio-de-salud-minsa
**Estonia**	Republic of Estonia Health Board: URL https://opendata.digilugu.ee/opendata_covid19_test_county_all.csv
**The Netherlands**	Ministry of Health, Welfare and Sport: URL https://opendata.arcgis.com/datasets/1365a2d9cb344b67999dd825c99cb1a5_0.csv
**Norway**	Thomas, Haarstad, F., Manuel & YBK. Public COVID-19 Data for Norway (covid19data.no): URL https://raw.githubusercontent.com/thohan88/covid19-nor-data/master/data/01_infected/msis/municipality.csv
**United Kingdom**	UK Health Security Agency: URL https://coronavirus.data.gov.uk/api/v2/data?areaType=ltla&metric=newCasesByPublishDate&format=csv
**Czech Republic**	National Health Information System, Regional Hygiene Station, Ministry of Health of the Czech Republic: URL https://onemocneni-aktualne.mzcr.cz/covid-19
**Germany**	Robert Koch Institut: URL https://www.arcgis.com/sharing/rest/content/items/f10774f1c63e40168479a1feb6c7ca74/data
**Romania**	Dénes Csala Data Consulting: URL https://raw.githubusercontent.com/denesdata/roem/master/data/time_series_ro_counties_daily.csv
**Switzerland**	Federal Office of Public Health FOPH: URL https://www.covid19.admin.ch/en/overview
**Italy**	Department of Civil Protection: URL https://github.com/pcm-dpc/COVID-19
**Spain**	Spain Ministry of Health and National Epidemiology Center (CNE) URL: https://cnecovid.isciii.es/covid19/resources/casos_hosp_uci_def_sexo_edad_provres.csv
**South Africa**	South Africa Department of Health: URL https://sacoronavirus.co.za/
**Japan**	NHK News: https://www3.nhk.or.jp/n-data/opendata/coronavirus/nhk_news_covid19_prefectures_daily_data.csv
**Kuwait**	Kuwait Ministry of Health :URL: https://corona.e.gov.kw/en

**The Philippines**	Phillipines Department of Health: URL https://doh.gov.ph/
**Singapore**	Data from Hopkins tracker, which lists: Singapore Ministry of Health: URL https://www.moh.gov.sg/covid-19
**Australia**	
Melbourne	Data from Hopkins tracker, which lists: Government Department of Health: URL https://www.health.gov.au/news/coronavirus-update-at-a-glance<br># COVID Live: URL https://www.covidlive.com.au/
Sydney	Data from Hopkins tracker, which lists: Government Department of Health: URL https://www.health.gov.au/news/coronavirus-update-at-a-glance<br># COVID Live: URL https://www.covidlive.com.au/
**Malaysia**	https://raw.githubusercontent.com/MoH-Malaysia/covid19-public/main/epidemic/cases_state.csv
**South Korea**	https://www.data.go.kr

Table S2. List of the 439 cities included in the studies, with cumulative COVID-19 cases and mean levels of meteorological variables and Oxford Government Index.

City Name	Country	COVID-19 cases	Daily mean temperature [°C]	Daily mean RH [%]	Daily mean AH [g/m^3]	Daily mean UV [W/m^3]	Daily mean Precipitations [mm]	Daily OXGRT SI Mean (%)
Brisbane	Australia	165380	19,5	73,4	12,8	186,8	3,6	54,6
Belém	Brazil	132830	26,7	81,5	21,0	209,7	7,1	56,4
Belo Horizonte	Brazil	682427	20,9	65,4	12,1	217,3	2,7	56,4
Brasília	Brazil	5686	21,6	60,7	11,6	228,2	2,8	55,2
Cuiabá	Brazil	155272	16,9	81,9	12,1	177,1	3,6	56,3
Curitiba	Brazil	196808	26,9	61,1	15,9	226,0	2,2	56,3
Fortaleza	Brazil	366853	26,9	75,1	19,6	221,1	2,8	56,4
Goiânia	Brazil	27695	23,3	57,9	12,2	229,0	2,7	54,4
João Pessoa	Brazil	173477	26,0	78,4	19,4	223,2	3,2	56,3
Maceió	Brazil	125192	25,3	78,7	18,8	200,1	4,6	56,2
Manaus	Brazil	304190	26,5	84,5	21,5	194,4	8,3	56,3
Natal	Brazil	140244	26,1	77,3	19,3	217,6	3,6	56,4
Porto Alegre	Brazil	299049	19,1	76,1	12,9	183,3	3,6	56,3
Recife	Brazil	265263	25,8	77,0	18,9	207,2	3,4	56,3
Salvador	Brazil	323547	25,2	79,4	18,9	203,2	4,3	56,3
São Luís	Brazil	1800	27,0	79,3	20,8	209,4	6,2	55,6
São Paulo	Brazil	1190287	19,2	76,6	12,9	196,3	3,1	55,6
Teresina	Brazil	124466	27,7	66,5	17,9	229,4	3,1	56,2
Vitória	Brazil	136205	23,1	79,1	16,7	205,5	2,8	56,3
Calgary Zone	Canada	236127	4,7	61,0	4,8	173,9	1,6	60,7
Edmonton Zone	Canada	189959	4,1	68,3	5,4	165,8	1,6	60,9
Vancouver Health Service Delivery Area	Canada	48314	9,9	79,4	7,8	149,1	6,7	60,6
Araucanía	Chile	85091	11,1	77,7	7,9	173,7	3,4	62,0
Región Metropolitana	Chile	122550	14,6	58,4	7,4	233,8	1,3	62,0
Valparaíso	Chile	79018	13,1	80,5	9,3	219,8	0,9	61,6
Prague	Czech Republic	528114	10,5	71,5	7,5	147,6	2,2	46,1
Tallinn	Estonia	284833	7,9	76,6	6,9	132,0	2,0	37,1
Berlin	Germany	1139316	11,1	71,0	7,5	137,0	1,7	53,1
Bremen	Germany	203241	10,9	76,1	8,0	134,7	2,3	53,4
Dortmund	Germany	206258	11,3	75,3	8,0	138,3	2,7	53,5
Dresden	Germany	240034	10,6	73,1	7,6	142,2	2,1	53,6
Duesseldorf	Germany	225196	11,9	74,3	8,2	140,9	2,6	53,6
Frankfurt	Germany	286766	11,7	71,2	7,7	146,5	2,0	53,5
Hamburg	Germany	696534	10,7	75,7	7,8	133,9	2,1	53,2
Koeln	Germany	404048	11,6	73,5	7,9	141,9	2,6	53,4
Leipzig	Germany	238384	11,1	71,8	7,6	142,6	1,9	53,6
Muenchen	Germany	612146	9,7	74,6	7,4	155,6	3,1	53,2
Stuttgart	Germany	219039	10,9	72,5	7,6	153,8	2,4	53,5
Ancona	Italy	178309	16,4	69,8	10,3	194,0	1,6	60,4
Bari	Italy	444627	17,7	69,2	11,0	203,3	1,5	60,3
Bologna	Italy	406276	15,7	65,1	9,0	184,4	1,7	60,4

Brescia	Italy	397754	14,1	71,3	9,3	174,1	2,9	60,4
Brindisi	Italy	131195	18,6	63,7	10,4	207,1	1,5	60,3
Cagliari	Italy	123965	18,6	68,8	11,3	213,5	1,2	60,1
Catania	Italy	329741	19,4	68,8	11,8	209,0	1,5	60,4
Firenze	Italy	348428	14,5	70,9	9,3	183,7	2,4	60,4
Frosinone	Italy	160233	15,7	71,1	9,8	197,6	3,0	60,2
Genova	Italy	272239	13,4	75,1	9,4	165,9	3,1	60,4
Latina	Italy	208818	17,1	74,6	11,4	200,8	2,4	60,3
Milano	Italy	1048785	14,4	68,0	8,9	175,5	3,0	60,4
Napoli	Italy	1132331	17,2	72,1	11,2	190,4	3,0	60,4
Padova	Italy	409575	14,9	71,1	9,7	179,5	2,5	60,4
Palermo	Italy	366649	18,0	71,9	11,5	215,2	1,7	60,4
Pisa	Italy	157257	16,2	71,9	10,6	190,9	2,8	60,4
Rieti	Italy	58633	12,8	68,0	7,8	192,8	2,5	60,1
Roma	Italy	1472858	16,9	69,8	10,5	198,6	2,2	60,4
Taranto	Italy	191406	18,6	66,7	11,0	207,8	1,3	60,1
Torino	Italy	745069	13,4	67,0	8,3	168,1	2,2	60,4
Trieste	Italy	97826	13,0	70,0	8,6	177,2	3,2	60,4
Venezia	Italy	354776	15,2	73,5	10,3	166,0	2,8	60,4
Viterbo	Italy	91432	15,2	69,9	9,4	197,8	2,3	60,3
Aichi	Japan	888268	15,7	74,8	11,6	175,3	5,2	43,3
Chiba	Japan	742432	16,6	76,8	12,3	167,8	5,1	43,4
Fukuoka	Japan	838039	16,9	77,2	12,6	172,3	5,3	43,4
Hokkaido	Japan	572859	8,9	79,6	8,4	167,2	3,3	43,0
Hyogo	Japan	722879	15,9	76,4	11,8	178,2	5,0	43,3
Kanagawa	Japan	1239953	16,1	76,2	11,9	164,4	4,8	43,4
Kyoto	Japan	368590	15,3	77,9	11,6	171,2	5,1	43,4
Osaka	Japan	1365742	16,6	74,3	12,0	175,2	4,8	43,2
Saitama	Japan	876266	15,7	75,0	11,6	161,3	4,3	43,4
Tokyo	Japan	2414005	15,9	76,2	11,9	161,9	4,2	43,3
Kuwait	Kuwait	713588	27,8	39,4	10,2	236,5	0,2	57,5
Area Metropolitana Monterrey	Mexico	346678	22,5	59,1	12,3	242,4	1,5	51,7
Zona Metropolitana Ciudad Juarez	Mexico	52538	20,5	29,8	5,5	266,8	0,5	51,7
Zona Metropolitana Guadalajara	Mexico	195099	21,9	51,2	9,8	261,0	2,7	50,8
Zona Metropolitana Laguna	Mexico	59091	20,1	38,6	6,8	274,4	0,9	51,2
Zona Metropolitana Leon	Mexico	136787	19,8	47,4	8,1	263,0	2,0	51,2
Zona Metropolitana Puebla	Mexico	171432	17,1	58,6	8,6	259,2	2,9	50,9
Zona Metropolitana Queretaro	Mexico	139112	18,8	52,9	8,5	258,1	1,8	51,3
Zona Metropolitana San Luis	Mexico	141342	17,9	54,6	8,4	259,3	1,6	51,4
Zona Metropolitana Tijuana	Mexico	69673	17,0	70,8	10,5	249,6	0,8	51,2
Zona Metropolitana Valle de Mexico	Mexico	2322352	16,4	53,6	7,5	255,7	1,6	50,3
Zona Metropolitana Valled de Toluca	Mexico	70222	13,2	64,0	7,4	247,6	3,9	51,9
Amsterdam	Netherland	392669	11,7	78,3	8,6	146,2	2,3	49,9
Den Haag	Netherland	214877	11,9	77,0	8,6	152,3	2,3	50,3

Eindhoven	Netherland	91416	12,1	73,8	8,2	143,1	2,3	50,2
Rotterdam	Netherland	284196	11,9	76,9	8,5	147,1	2,3	50,0
Utrecht	Netherland	155172	11,7	77,1	8,4	142,6	2,3	50,0
Oslo	Norway	197687	7,9	75,2	6,7	129,1	2,5	41,1
Apurimac	Peru	43066	7,3	68,3	5,4	239,7	3,8	68,6
Arequipa	Peru	225428	13,9	52,7	6,3	263,9	2,2	69,2
Ayacucho	Peru	52022	11,2	70,8	7,2	235,4	2,9	68,5
Cajamarca	Peru	107064	12,8	76,8	8,6	221,6	8,4	68,7
Cusco	Peru	130735	8,1	66,4	5,5	218,8	3,1	69,2
Huancavelica	Peru	27804	4,2	76,5	5,0	243,7	4,2	68,6
Huanuco	Peru	56236	14,4	78,3	9,7	213,7	4,1	68,9
Ica	Peru	99217	21,3	60,0	11,4	262,7	0,2	69,0
Junin	Peru	139733	3,9	80,5	5,1	241,1	4,3	69,1
La Libertad	Peru	147961	9,0	75,7	6,7	235,7	5,7	69,3
Lambayeque	Peru	103701	21,3	73,0	13,8	244,6	0,5	69,2
Lima	Peru	1706119	18,6	79,5	12,9	243,3	0,5	68,9
Loreto	Peru	61536	25,5	88,1	21,2	186,8	8,3	69,4
Piura	Peru	139494	23,7	62,0	13,5	251,8	0,6	69,0
Puno	Peru	64180	8,4	55,9	4,8	274,3	3,3	68,7
San Martin	Peru	66237	23,2	86,3	18,2	204,8	6,1	69,0
Tacna	Peru	51099	17,4	62,7	9,4	259,1	0,5	68,6
Ucayali	Peru	42553	25,8	84,2	20,6	203,8	6,0	68,9
Cebu City	Philippines	58135	26,2	82,3	20,7	202,8	8,7	67,4
Davao City	Philippines	141329	26,4	81,8	20,8	195,5	5,6	67,2
Manila City	Philippines	73836	27,5	77,2	20,8	203,9	5,9	67,3
Quezon City	Philippines	232331	27,8	77,2	21,2	204,4	6,6	67,2
Brasov	Romania	109880	8,7	74,2	7,1	157,6	2,3	52,2
Bucuresti	Romania	565570	13,1	67,6	8,2	167,9	1,8	52,2
Cluj	Romania	166565	9,1	75,2	7,4	155,2	2,3	52,2
Constanta	Romania	117217	13,4	72,8	9,3	168,6	1,7	52,2
Dolj	Romania	74686	13,3	64,5	7,8	170,8	1,7	52,2
Galati	Romania	65332	13,3	65,4	8,1	164,3	1,3	52,2
Iasi	Romania	113197	11,6	67,3	7,6	154,7	1,5	52,2
Timis	Romania	154915	12,8	68,4	8,2	165,0	2,0	52,2
Singapore	Singapore	1865432	27,0	82,3	21,7	190,7	7,3	48,7
City of Cape Town	South Africa	414972	16,1	74,7	10,4	202,8	1,6	52,3
A Coruna	Spain	240648	13,7	81,5	9,9	155,0	3,0	57,1
Ãvila	Spain	37245	11,3	65,2	6,5	193,9	1,7	57,3
Albacete	Spain	84476	14,5	60,4	7,4	201,3	1,4	57,1
Alicante	Spain	417664	17,7	67,9	10,7	191,9	1,2	56,8
Almeria	Spain	131379	17,9	61,9	9,6	206,0	0,8	57,4
Araba	Spain	76119	11,1	79,0	8,2	158,6	2,5	56,7
Asturias	Spain	200878	12,3	82,1	9,3	164,7	3,6	57,1
Badajoz	Spain	163368	17,6	59,1	8,6	201,4	1,3	57,3
Barcelona	Spain	1421266	15,5	76,4	10,7	168,6	1,5	55,9
Bizkaia	Spain	272291	13,6	78,3	9,6	155,2	3,6	56,8
Burgos	Spain	90452	10,9	71,7	7,2	176,0	1,9	57,0

Caceres	Spain	85166	16,8	59,4	8,2	200,1	1,5	57,1
Cadiz	Spain	191632	18,7	65,9	10,7	197,5	1,4	57,3
Cordoba	Spain	141164	18,3	55,7	8,4	208,6	1,2	57,3
Cantabria	Spain	112518	14,0	79,2	9,9	149,2	3,4	57,1
Castellana	Spain	134006	17,0	72,5	11,1	190,0	1,7	57,3
Ceuta	Spain	17364	17,5	77,3	11,9	180,0	1,7	55,5
Ciudad Real	Spain	107629	15,9	54,7	7,0	203,8	1,2	56,8
Cuenca	Spain	48150	11,8	63,5	6,7	193,6	1,9	56,9
Gipuzkoa	Spain	210834	13,5	78,4	9,6	154,1	4,4	57,2
Girona	Spain	206023	14,5	75,1	9,9	168,4	2,5	56,9
Granada	Spain	173477	16,0	56,8	7,5	213,1	1,5	57,1
Guadalajara	Spain	62273	13,7	59,1	6,8	192,6	1,6	56,8
Huelva	Spain	83723	19,0	62,7	10,2	214,3	1,2	57,5
Huesca	Spain	63523	14,1	64,8	8,0	190,2	1,8	57,5
Illes Balears	Spain	261004	17,8	72,1	11,4	199,8	1,3	57,0
Jaén	Spain	113567	16,7	57,4	7,9	208,6	1,6	57,3
La Rioja	Spain	86305	12,8	71,7	8,2	167,5	1,8	56,7
Las Palmas	Spain	155350	19,4	75,5	12,8	235,7	0,7	57,2
Leon	Spain	106861	11,1	68,6	7,0	176,2	1,7	57,3
Lleida	Spain	125577	15,7	61,0	8,3	190,7	1,2	57,2
Lugo	Spain	63078	11,6	81,1	8,7	160,7	2,9	57,3
Malaga	Spain	273678	14,6	57,1	6,9	195,9	1,4	56,8
Madrid	Spain	1519220	17,6	71,5	11,0	211,0	1,1	55,6
Melilla	Spain	19531	18,1	70,8	11,3	189,3	0,9	55,6
Murcia	Spain	341150	17,8	61,3	9,6	206,1	1,1	57,0
Navarra	Spain	201175	11,5	77,6	8,3	162,9	2,4	56,9
Ourense	Spain	70158	12,9	77,9	9,1	167,6	2,7	57,3
Palencia	Spain	50307	12,1	65,6	7,0	185,3	1,5	57,6
Pontevedra	Spain	213447	13,7	80,3	9,8	174,0	4,0	57,2
Salamanca	Spain	81489	12,9	63,9	7,1	191,1	1,5	57,1
Santa Cruz de Tenerife	Spain	131793	18,7	75,2	12,3	231,5	0,7	57,0
Segovia	Spain	38759	11,8	66,0	6,9	190,2	2,1	57,3
Sevilla	Spain	287489	18,9	60,1	9,7	211,6	1,3	57,1
Soria	Spain	23615	10,8	67,3	6,7	182,5	1,9	57,3
Tarragona	Spain	215436	16,3	69,1	10,1	178,6	1,5	57,1
Teruel	Spain	38918	11,5	64,7	6,8	192,7	1,4	57,6
Toledo	Spain	165122	15,6	58,6	7,5	200,4	1,3	56,8
Valencia	Spain	678158	17,5	69,3	10,8	193,3	1,7	56,7
Valladolid	Spain	146556	12,6	65,0	7,1	187,6	1,5	57,3
Zamora	Spain	41505	13,2	63,9	7,2	189,2	1,5	57,4
Zaragoza	Spain	245961	15,5	59,7	8,0	190,1	1,2	56,8
Basel-Stadt	Switzerland	95866	11,6	73,1	8,0	160,2	2,6	41,6
Bern	Switzerland	450196	9,3	74,2	7,2	166,4	3,5	41,7
Geneva	Switzerland	225760	11,1	73,4	7,8	169,7	4,1	41,6
Lucerne	Switzerland	188017	10,5	75,5	7,8	163,4	4,4	41,9
Sankt Gallen	Switzerland	226388	8,3	76,7	7,1	161,0	4,5	41,9
Ticino	Switzerland	164862	11,2	68,3	7,6	166,8	4,1	41,6

Vaud	Switzerland	345448	12,1	74,4	8,5	166,9	3,6	41,6
Zurich	Switzerland	703917	10,5	73,4	7,6	163,0	3,1	41,6
Barnsley	United Kingdom	91277	10,1	80,1	7,8	121,7	2,5	53,5
Basildon	United Kingdom	69076	11,5	78,1	8,4	140,8	2,0	53,4
Basingstoke and Deane	United Kingdom	64176	11,0	78,8	8,1	134,5	2,1	53,4
Bedford	United Kingdom	68306	11,0	77,3	8,0	129,2	2,0	53,5
Birmingham	United Kingdom	346741	10,3	78,5	7,8	124,6	2,2	53,5
Blackburn with Darwen	United Kingdom	55268	9,5	82,0	7,8	124,2	3,7	53,5
Blackpool	United Kingdom	51180	10,8	79,6	8,1	130,4	4,0	53,5
Brighton and Hove	United Kingdom	103151	11,5	79,0	8,5	140,1	2,2	53,5
Bristol, City of	United Kingdom	179000	11,0	79,8	8,3	130,5	2,4	53,5
Burnley	United Kingdom	32142	8,6	84,2	7,5	111,6	3,8	51,9
Cambridge	United Kingdom	49895	11,0	78,1	8,1	122,7	2,1	51,9
Chelmsford	United Kingdom	65790	11,2	78,1	8,2	139,2	2,0	53,4
Cheltenham	United Kingdom	40522	10,5	80,0	8,0	120,1	2,3	51,9
Chesterfield	United Kingdom	36305	9,9	80,1	7,7	122,9	2,4	53,4
Colchester	United Kingdom	71016	11,3	78,9	8,4	139,4	2,0	53,4
Coventry	United Kingdom	115921	10,6	78,9	8,0	125,9	2,2	53,5
Crawley	United Kingdom	43247	11,1	79,6	8,3	134,1	2,2	51,9
Derby	United Kingdom	89838	10,3	79,4	7,9	124,3	2,2	53,5
Doncaster	United Kingdom	110977	10,4	78,2	7,8	125,3	2,2	53,5
Eastbourne	United Kingdom	35337	11,9	80,1	8,8	142,3	2,4	51,8
Exeter	United Kingdom	47425	11,2	82,3	8,6	131,2	2,3	51,9
Gloucester	United Kingdom	45733	10,9	79,0	8,1	127,8	2,2	53,4
Hastings	United Kingdom	32381	11,5	79,7	8,6	136,7	2,3	51,9
Ipswich	United Kingdom	49622	11,1	78,9	8,3	139,3	2,1	53,4
Kingston upon Hull, City of	United Kingdom	94036	10,5	79,3	8,0	132,3	2,2	53,5
Leicester	United Kingdom	118526	10,5	78,8	7,9	126,2	2,2	53,5
Lincoln	United Kingdom	35565	10,6	78,2	7,9	127,2	2,2	53,4
Liverpool	United Kingdom	179760	10,7	79,6	8,1	128,5	3,2	53,5
London	United Kingdom	3003884	11,3	77,8	8,2	134,3	2,1	53,5
Luton	United Kingdom	75529	10,7	77,9	7,9	130,3	2,1	53,5

Maidstone	United Kingdom	61447	11,2	78,8	8,3	146,6	2,2	53,4
Manchester	United Kingdom	190877	10,0	81,6	7,9	120,0	3,1	53,5
Mansfield	United Kingdom	39911	9,9	79,7	7,7	117,2	2,2	51,9
Medway	United Kingdom	98691	11,1	78,7	8,2	144,1	2,2	53,5
Milton Keynes	United Kingdom	103904	10,8	78,0	8,0	129,4	2,1	53,5
Northampton	United Kingdom	82110	10,6	78,4	7,9	127,3	2,1	53,4
Norwich	United Kingdom	49451	11,0	78,6	8,2	142,3	2,1	53,4
Nottingham	United Kingdom	109818	10,4	78,2	7,8	124,8	2,1	53,5
Oxford	United Kingdom	57101	10,9	78,7	8,1	129,3	2,1	53,4
Peterborough	United Kingdom	77455	11,1	77,2	8,0	128,2	2,1	53,5
Plymouth	United Kingdom	98032	11,5	81,6	8,7	146,6	2,7	53,5
Preston	United Kingdom	51945	10,2	80,9	8,0	126,7	3,8	53,4
Reading	United Kingdom	61371	11,0	78,4	8,1	132,7	2,1	53,5
Sheffield	United Kingdom	196595	9,8	80,7	7,8	122,2	2,5	53,5
Slough	United Kingdom	53010	11,3	78,1	8,2	132,6	2,0	53,5
Southend-on-Sea	United Kingdom	64773	11,8	78,5	8,6	143,9	2,2	53,2
Stoke-on-Trent	United Kingdom	91629	10,0	80,2	7,8	123,2	2,5	53,5
Sunderland	United Kingdom	101387	10,1	77,7	7,6	126,3	2,1	53,5
Swindon	United Kingdom	79339	10,8	79,4	8,1	131,0	2,2	53,5
Thanet	United Kingdom	48348	11,7	79,2	8,6	144,0	2,0	53,4
Warrington	United Kingdom	81024	10,3	79,8	7,9	125,8	3,1	53,5
Wigan	United Kingdom	118319	10,2	79,7	7,9	126,0	3,1	53,5
Worcester	United Kingdom	35939	10,9	78,2	8,0	126,1	2,1	53,4
York	United Kingdom	71296	10,0	79,0	7,7	125,0	2,2	53,5
Akron	United States	113651	10,8	69,4	8,1	168,9	3,3	57,3
Albany	United States	62428	10,1	70,3	8,0	164,2	3,4	57,3
Albuquerque	United States	144816	15,2	32,9	4,4	249,4	0,5	57,4
Allentown	United States	85428	11,5	69,6	8,5	170,8	3,4	57,4
Anaheim	United States	509438	17,7	63,4	9,8	235,8	0,7	55,2
Anchorage	United States	82182	2,8	78,7	5,4	110,9	2,8	56,9
Ann Arbor	United States	64189	10,0	68,0	7,6	171,8	2,5	57,2

Annandale	United States	150808	14,1	67,8	9,6	177,6	3,3	57,4
Atlanta	United States	455255	16,0	72,3	11,0	182,7	4,0	53,7
Atlantic City	United States	67574	13,8	72,0	10,0	182,0	3,4	58,0
Augusta	United States	62969	17,8	69,5	11,6	187,0	3,4	53,7
Austin	United States	97229	20,4	64,8	12,6	197,4	2,7	57,1
Aztec	United States	39027	12,2	37,3	3,8	248,6	0,6	58,1
Bakersfield	United States	238718	19,8	42,3	6,9	244,5	0,6	57,6
Baltimore	United States	266765	13,3	69,7	9,5	173,9	3,6	57,7
Bangor	United States	27787	8,3	70,6	7,3	161,6	2,9	57,6
Barnstable	United States	43752	11,3	75,6	8,9	177,3	3,3	57,1
Bath	United States	22434	17,4	73,9	12,3	184,4	3,7	58,1
Baton Rouge	United States	96392	20,0	76,7	14,5	191,4	4,3	57,4
Beaver Dam	United States	20394	9,0	68,8	7,5	171,1	2,3	57,1
Birmingham	United States	235899	17,2	71,7	11,7	190,0	4,2	57,3
Boise City	United States	130188	11,1	49,4	4,8	207,9	1,1	57,4
Boston	United States	213163	10,5	69,6	8,1	167,1	3,0	56,6
Boulder	United States	65139	6,5	55,8	4,6	224,4	1,8	57,1
Brownsville	United States	34931	24,0	72,9	16,9	210,5	2,0	58,0
Buffalo	United States	229710	9,9	71,4	7,9	166,1	3,0	57,4
Burlington	United States	29442	10,5	68,4	7,9	167,2	3,0	57,3
Canton	United States	80342	11,0	69,1	8,2	169,0	3,2	58,1
Carlisle	United States	51599	12,3	68,1	8,7	171,6	3,2	58,1
Cedar Rapids	United States	31667	11,0	68,7	8,4	180,0	2,6	61,5
Charleston	United States	135021	19,5	76,5	14,0	200,4	3,6	57,1
Charlotte	United States	300514	16,3	69,1	10,7	187,9	3,4	57,1
Chattanooga	United States	101116	16,0	72,1	11,0	184,3	3,8	57,6
Chicago	United States	1169438	10,0	73,4	8,2	177,2	3,0	56,6
Cincinnati	United States	216054	12,9	68,1	9,1	175,1	3,5	57,3
Cleveland	United States	241821	10,9	72,6	8,5	176,1	3,2	57,1
Colorado Springs	United States	184746	8,6	45,9	4,3	227,5	1,2	57,1
Columbia	United States	183438	18,0	69,5	11,9	190,2	3,3	57,1

Columbus	United States	296689	11,7	69,3	8,6	172,5	3,3	56,6
Corpus Christi	United States	19790	22,6	74,1	16,2	188,0	2,6	57,2
Dallas	United States	116168	19,2	63,8	11,8	197,0	2,9	56,9
Dayton	United States	158958	12,0	69,0	8,7	175,6	3,1	57,6
Daytona Beach	United States	75961	22,5	74,8	15,8	187,2	3,3	57,6
Denver	United States	579744	9,1	48,0	4,6	221,0	1,3	56,6
Detroit	United States	275055	10,4	68,4	7,9	175,3	2,3	56,6
Dover	United States	44160	14,3	68,3	9,8	180,3	3,8	58,1
Durham	United States	76470	16,0	70,1	10,9	185,8	3,6	57,1
East St. Louis	United States	63322	13,9	66,5	9,5	181,1	3,4	57,8
El centro	United States	59039	24,8	24,6	5,8	260,2	0,1	58,0
El Paso	United States	65175	19,1	29,6	5,0	260,4	0,4	57,9
Elizabeth	United States	127145	12,1	69,5	8,8	168,9	3,5	56,8
Elkhart	United States	55333	10,7	70,5	8,2	172,0	3,2	58,0
Erie	United States	59569	10,3	75,4	8,6	171,1	3,5	57,3
Essex	United States	214696	11,8	69,1	8,6	168,8	3,6	56,6
Eugene	United States	58741	11,6	73,6	7,7	168,6	3,5	56,4
Evansville	United States	57924	14,8	66,4	9,9	183,4	3,6	57,2
Everett	United States	146330	10,0	79,1	7,8	139,9	5,7	56,6
Fargo	United States	59398	7,0	63,3	6,2	175,1	1,8	58,1
Fayetteville	United States	93350	17,3	69,1	11,5	188,0	3,5	57,9
Flint	United States	72245	9,5	67,3	7,3	169,1	2,4	56,6
Fort Lauderdale	United States	473704	24,6	75,2	17,6	201,8	3,9	56,6
Fort Myers	United States	112020	23,7	74,4	16,7	212,6	3,4	57,1
Fort Pierce	United States	56612	23,8	75,2	16,9	187,0	3,8	57,5
Fort Wayne	United States	106158	11,0	68,8	8,2	174,9	3,0	57,7
Fort Worth	United States	28281	19,3	60,4	11,3	199,3	2,2	56,5
Fresno	United States	246898	19,8	44,2	7,1	238,5	0,8	57,5
Gainesville	United States	52989	21,4	73,7	14,8	194,7	3,2	58,0
Gary	United States	104218	10,7	71,7	8,4	178,4	3,3	57,5
Gettysburg	United States	25085	13,0	67,6	9,1	174,4	3,1	58,0

Grand Haven	United States	57818	9,7	73,7	8,1	176,8	2,6	58,1
Grand Junction	United States	43166	10,9	42,1	3,9	221,4	1,0	55,8
Grand Rapids	United States	118117	9,8	68,1	7,5	172,6	2,7	57,4
Green Bay	United States	56003	8,4	68,2	7,2	171,2	2,4	58,0
Greensboro	United States	138942	15,3	69,3	10,3	187,8	3,6	57,9
Greensburg	United States	80986	11,2	70,2	8,3	167,2	3,6	57,8
Greenville	United States	146586	15,8	70,6	10,6	188,9	3,9	57,4
Harrisburg	United States	58547	12,3	67,8	8,7	170,9	3,1	57,8
Hickory	United States	58304	15,6	69,6	10,5	186,5	3,5	57,1
Holland	United States	57818	9,8	72,2	7,9	175,9	2,8	58,1
Honolulu	United States	25177	24,0	71,5	15,8	237,5	1,1	57,7
Houston	United States	191471	20,8	74,5	14,8	189,3	3,6	56,7
Indianapolis	United States	285198	12,1	67,6	8,6	175,7	3,1	57,0
Iowa city	United States	20024	11,9	68,6	8,8	181,6	2,8	62,0
Jacksonville	United States	146209	21,1	75,9	15,0	195,5	3,4	57,1
Jersey City	United States	146629	12,2	70,3	9,0	170,0	3,4	56,6
Kalamazoo	United States	41075	10,1	70,9	7,9	172,0	2,9	58,1
Kansas City	United States	42564	13,4	65,6	9,1	184,7	3,0	57,7
Kenosha	United States	37616	9,1	75,4	7,9	172,7	2,5	57,6
Klamath Falls	United States	14157	8,4	58,7	4,9	190,4	1,4	54,5
Knoxville	United States	126849	14,5	70,9	9,9	183,7	3,7	57,6
La Porte	United States	26545	10,8	71,2	8,4	176,5	3,3	58,0
Lafayette	United States	59704	20,6	74,0	14,5	194,4	3,7	57,7
Lafayette (IN)	United States	50004	12,0	67,5	8,7	177,8	3,0	57,6
Lake Charles	United States	50939	20,5	76,1	14,9	197,0	4,3	57,8
Lakeland	United States	147823	22,7	73,8	15,7	201,7	3,5	57,4
Lancaster	United States	122864	12,6	68,3	8,9	173,0	3,3	57,5
Lansing	United States	68699	9,8	67,4	7,5	170,4	2,5	57,3
Las Vegas	United States	491968	20,4	24,1	4,1	251,2	0,2	56,6
Layton	United States	99573	9,7	52,2	4,8	224,0	1,1	57,6
Little Rock	United States	98860	16,9	70,3	11,4	188,3	4,1	57,6

Logan	United States	36225	11,2	70,5	8,5	159,5	3,0	57,1
Los Angeles	United States	2381601	17,5	57,5	8,7	235,4	0,7	53,7
Louisville	United States	66485	13,6	70,5	9,8	176,9	3,6	57,4
Macon	United States	28828	18,2	68,3	11,7	188,0	3,4	53,7
Madison	United States	69719	9,1	68,6	7,5	173,0	2,4	57,8
Madison (WI)	United States	98301	9,0	68,6	7,4	172,9	2,4	57,3
Mcallen	United States	43559	24,4	64,5	15,3	218,1	1,8	57,6
Medford	United States	42368	11,3	63,6	6,4	195,4	2,1	56,1
Melbourne	United States	73256	23,4	75,7	16,7	191,1	3,5	57,6
Melville	United States	371135	12,1	73,1	9,2	176,7	3,5	56,9
Memphis	United States	220712	16,5	70,1	11,2	188,9	4,0	57,3
Mercer	United States	23188	10,0	72,4	8,2	157,9	3,6	56,1
Miami	United States	924750	24,9	75,0	17,9	208,6	3,7	56,6
Middlesex	United States	179307	12,3	68,4	8,8	169,6	3,6	57,0
Middletown	United States	89617	10,9	70,1	8,4	168,7	3,5	57,9
Milwaukee	United States	159363	8,8	73,1	7,6	172,6	2,4	57,1
Minneapolis	United States	388999	8,0	65,7	6,8	173,2	2,4	57,1
Mobile	United States	108133	20,1	77,3	14,7	185,0	4,8	57,5
Modesto	United States	136428	18,6	48,2	7,3	235,9	0,7	58,1
Monroe	United States	47437	18,9	71,2	12,8	192,5	4,0	57,8
Montgomery	United States	51306	18,9	69,8	12,4	196,6	3,3	58,1
Muncie	United States	25195	11,8	68,1	8,5	178,1	3,0	57,9
Muskegon	United States	26468	9,8	72,6	8,0	176,8	2,5	57,8
Myrtle Beach	United States	94361	18,9	74,4	13,3	194,6	3,7	58,1
Nampa	United States	61924	12,8	45,0	4,8	207,3	0,8	57,6
Nashville	United States	186615	15,2	72,4	10,8	180,9	4,1	57,1
New hHaven	United States	96086	11,6	70,5	8,7	175,2	3,6	57,1
New London	United States	76737	11,4	71,7	8,7	176,8	3,4	58,1
New Orleans	United States	180572	20,9	77,2	15,2	192,5	4,8	56,7
New York	United States	367680	12,1	70,3	8,9	168,8	3,5	55,9
Newark	United States	173216	12,1	69,5	8,8	168,9	3,5	56,7

Newburgh	United States	110897	10,6	69,7	8,2	167,9	3,4	56,9
Niles	United States	26507	10,6	71,0	8,2	173,0	3,3	58,1
Norfolk	United States	40602	16,3	71,9	11,3	186,4	3,4	57,7
Oakland	United States	238545	14,5	65,9	8,2	224,9	0,7	57,1
Ocala	United States	56152	21,8	73,5	15,0	198,6	3,2	57,8
Ogden	United States	70334	9,8	51,7	4,8	223,0	1,1	57,4
Oklahoma City	United States	176155	16,5	60,4	9,8	204,8	2,4	57,3
Omaha	United States	76603	11,5	62,9	8,0	190,5	2,3	57,8
Orlando	United States	295198	22,5	75,1	15,9	195,6	3,6	57,2
Ottawa	United States	28848	11,3	67,6	8,4	175,8	3,0	57,5
Palm beach	United States	303924	24,0	75,8	17,2	197,9	4,1	57,1
Paterson	United States	128012	11,6	68,8	8,5	168,8	3,6	56,6
Pensacola	United States	52468	20,5	76,9	14,9	199,4	4,7	57,8
Philadelphia	United States	404410	13,1	67,3	9,1	171,6	3,4	57,1
Phoenix	United States	1297197	23,8	27,4	5,9	251,4	0,5	57,1
Pittsburgh	United States	260698	11,3	70,5	8,5	166,6	3,6	57,2
Plymouth	United States	125580	10,0	68,2	7,6	171,5	2,5	57,1
Port Arthur	United States	11259	21,0	78,6	15,7	195,2	4,2	56,5
Portage	United States	38842	11,7	73,2	7,7	161,4	3,6	58,1
Portland	United States	271589	11,5	73,3	7,6	160,4	3,5	56,9
Portland (ME)	United States	51987	9,2	71,6	7,6	160,1	3,3	57,1
Provo	United States	190116	10,2	51,0	4,8	229,4	0,9	57,7
Raleigh	United States	275673	16,5	69,1	11,0	185,9	3,6	57,3
Reading	United States	102103	11,8	69,2	8,6	170,9	3,4	57,5
Reno	United States	93256	11,3	47,1	4,4	238,0	1,0	57,6
Richmond	United States	170072	15,3	68,7	10,3	181,7	3,5	57,6
Riverside	United States	582853	18,3	49,1	7,7	242,3	1,1	57,2
Rochester	United States	162947	9,9	70,6	7,8	163,8	3,0	57,5
Rockville	United States	110956	13,5	68,1	9,3	176,2	3,4	57,9
Sacramento	United States	276106	17,5	51,7	7,4	227,8	1,1	57,0
Salt Lake City	United States	321176	10,1	53,2	5,1	223,8	1,2	57,2

San Antonio	United States	165553	20,9	64,0	12,8	200,8	2,4	57,2
San Diego	United States	691545	17,0	69,0	10,2	240,9	0,7	56,9
San Francisco	United States	248034	13,8	72,5	8,7	220,3	0,8	56,7
San Jose	United States	295634	15,7	58,8	7,8	228,6	0,9	56,3
Santa Barbara	United States	82099	17,2	50,4	7,5	236,3	0,7	57,4
Sarasota	United States	126866	23,2	75,4	16,5	215,5	3,2	57,1
Scranton	United States	41957	9,7	70,7	7,8	167,8	3,1	57,6
Seattle	United States	345071	10,8	77,0	7,9	142,5	3,5	56,2
Sioux City	United States	21290	12,0	62,6	8,1	190,5	1,9	62,1
South bend	United States	72058	10,5	71,0	8,2	172,3	3,3	57,7
Spartanburg	United States	84214	16,3	69,2	10,7	188,9	3,5	57,9
Spokane	United States	118949	10,0	59,3	5,5	178,6	1,6	57,4
Springfield	United States	69939	13,9	69,0	9,6	184,4	3,8	58,2
Springfield (MA)	United States	136674	10,3	69,0	7,9	167,2	3,2	57,1
St. Charles	United States	89608	13,5	67,7	9,5	180,0	3,2	57,9
St. Louis	United States	254191	13,7	66,4	9,4	179,9	3,4	57,4
St. Petersburg	United States	137659	23,0	75,8	16,4	206,5	3,2	57,2
Stamford	United States	141467	11,5	71,3	8,7	170,1	3,4	57,0
State College	United States	36037	10,1	69,9	7,8	163,4	3,0	56,3
Steubenville	United States	13389	11,6	70,4	8,7	168,6	3,3	57,6
Stockton	United States	160315	17,0	53,4	7,6	233,1	0,9	57,3
Tacoma	United States	176686	10,8	77,5	7,9	147,0	3,6	57,0
Tallahassee	United States	51009	20,3	74,7	14,2	198,4	4,0	58,0
Tampa	United States	203779	22,8	74,5	16,0	202,1	3,4	57,1
Terre Haute	United States	28977	12,5	67,2	8,9	171,9	3,2	56,0
Toledo	United States	135406	11,0	68,9	8,1	173,5	2,6	57,3
Toms River	United States	158825	13,2	70,2	9,4	174,6	3,6	56,6
Topeka	United States	48587	13,9	63,5	9,2	189,4	2,8	57,3
Trenton	United States	178202	12,8	68,2	9,0	171,0	3,5	57,1
Tucson	United States	255552	22,5	29,4	6,0	255,8	0,6	57,4
Tulsa	United States	128118	16,2	65,1	10,4	192,9	3,5	57,3

Upper Marlboro	United States	125319	14,2	69,6	9,9	176,7	3,5	57,6
Vancouver	United States	85367	11,6	74,2	7,8	159,7	3,7	57,5
Ventura	United States	165666	17,2	61,9	9,3	245,3	0,7	57,4
Visalia	United States	133121	20,0	44,4	7,1	237,7	0,9	58,1
Washington	United States	52532	11,5	69,9	8,6	164,2	3,5	56,4
Wichita	United States	135370	14,6	60,4	8,9	201,4	2,4	57,7
Wilmington	United States	146257	13,4	69,8	9,6	174,1	3,5	57,7
Winston-Salem	United States	106971	15,3	68,5	10,1	186,9	3,6	57,5
Worcester	United States	207303	9,6	69,5	7,6	167,0	3,4	57,2
York	United States	118849	12,7	67,3	8,8	173,2	3,1	57,5
Youngstown	United States	44599	10,6	70,8	8,2	166,9	3,4	57,3

Table S3: Population included in the analysis relative to each countries' total population

**Country**	**Study Population**	**Number of cities**	**Total Population, year 2022**	**% Total population included in the Study**
Australia	1856353	1	26010000	7.1
Brazil	61690882	18	215300000	28.7
Canada	4471879	3	38930000	11.5
Chile	3720629	3	19600000	19.0
Czech Republic	583087	1	9042000	6.4
Estonia	537039	1	1349000	39.8
Germany	19549908	11	83800000	23.3
Italy	15651954	23	58940000	26.6
Japan	51012754	10	125100000	40.8
Kuwait	4270571	1	4260000	100.2
Mexico	47862723	11	127500000	37.5
Netherland	4607655	5	11690000	39.4
Norway	997500	1	5457000	18.3
Peru	21935550	18	34045000	64.4
Philippines	6372858	4	115600000	5.5
Romania	4004684	8	19050000	21.0
Singapore	3900000	1	5637000	69.2
South Africa	4040358	1	59890000	6.7
Spain	20726264	52	47780000	43.4
Switzerland	3190056	8	8776000	36.3
United Kingdom	28341792	54	66970000	42.3
United States	64511813	204	333300000	19.4
**MCC Total**	**373836306**	**439**	**1418026000**	**26.4**
**Global**			7951000000	4.7

Table S4: Associations (RRs and 95%CI) between meteorological variables and COVID-19 incidence. Sensitivity analysis considering a linear relationship.

	RRs	I^2
Mean temperature (5°C vs 17°C)	1.10 (1.03; 1.08)	75.5
RH (60% vs 70%)	1.02 (1.00; 1.05)	74.3
AH (5 g/m^3 vs 9 g/m^3)	1.16 (1.06; 1.27)	81.4
UV (100 W/m^2 vs 200 W/m^2)	0.97 (0.94; 1.00)	66.9
Daily precipitation (0 mm vs 5 mm)	1.02 (1.01; 1.04)	69.2

Table S5: Associations (RRs and 95%CI) between meteorological variables and COVID-19 incidence. Sensitivity Analysis: Vaccination coverage threshold 75%

	Vaccination coverage <75%	Vaccination coverage >75%
Mean temperature (5°C vs 17°C)	1.10 (0.87; 1.38)	2.25 (0.96; 5.26)
RH (60% vs 70%)	1.04 (1.02; 1.05)	-
AH (5 g/m^3 vs 9 g/m^3)	1.11 (1.01; 1.22)	-
UV (100 W/m^2 vs 200 W/m^2)	0.91 (0.68; 1.21)	-
Daily precipitation (0 mm vs 5 mm)	1.01 (0.99; 1.03)	-

Table S6: Associations (RRs and 95%CI) between meteorological variables and COVID-19 incidence. Sensitivity Analysis: Fully vaccinated

	Vaccination coverage <60%	Vaccination coverage > 60%
Mean temperature (5°C vs 17°C)	1.10 (0.91; 1.33)	2.37 (1.19; 4.75)
RH (60% vs 70%)	1.03 (1.01; 1.05)	1.00 (0.78; 1.27)
AH (5 g/m^3 vs 9 g/m^3)	1.15 (1.03; 1.29)	1.19 (0.75; 1.90)
UV (100 W/m^2 vs 200 W/m^2)	0.91 (0.67; 1.22)	1.10 (0.52; 2.31)
Daily precipitation (0 mm vs 5 mm)	1.01 (0.99; 1.02)	1.03 (0.98; 1.08)

Table S7: Associations (RRs and 95%CI) between meteorological variables and COVID-19 incidence. Sensitivity Analysis: Linear relationship among vaccination rates of 60%

	Vaccination coverage <60%	Vaccination coverage > 60%
Mean temperature (5°C vs 17°C)	1.14 (1.06; 1.22)	1.06 (0.98; 1.17)
RH (60% vs 70%)	1.03 (1.01; 1.06)	1.02 (0.99; 1.05)
AH (5 g/m^3 vs 9 g/m^3)	1.18 (1.07; 1.30)	1.16 (1.04; 1.29)
UV (100 W/m^2 vs 200 W/m^2)	0.99 (0.96; 1.03)	0.92 (0.87; 0.98)
Daily precipitation (0 mm vs 5 mm)	1.01 (1.00; 1.03)	1.03 (0.99; 1.07)

Table S8: Associations (RRs and 95%CI) between meteorological variables and COVID-19 incidence. Sensitivity Analysis: Linear relationship among dominant strains

	Initial (First Wave)	Delta	Omicron
Mean temperature (5°C vs 17°C)	1.13 (1.03; 1.24)	1.10 (1.03; 1.17)	1.31 (1.19; 1.45)
RH (60% vs 70%)	1.02 (0.99; 1.05)	1.01 (0.99; 1.03)	1.04 (1.02; 1.06)
AH (5 g/m^3 vs 9 g/m^3)	1.16 (1.05; 1.29)	1.11 (1.03; 1.19)	1.24 (1.14; 1.34)
UV (100 W/m^2 vs 200 W/m^2)	0.96 (0.93; 0.99)	0.98 (0.95; 1.01)	1.07 (1.05; 1.10)
Daily precipitation (0 mm vs 5 mm)	1.04 (1.01; 1.08)	1.00 (0.98; 1.01)	1.06 (1.04; 1.08)

Table S9: Correlation matrix for all the meteorological variables in the 439 cities.

	Temperature	RH	AH	Precipitation	UV
Temperature		-0.18	0.81	0.27	-0.01
RH			0.42	0.19	0.57
AH				-0.56	0.28
Precipitation					-0.32
UV					
